# Supplementary material for: Gustatory interface for operative assessment and taste decoding in patients with tongue cancer
Source: Nat Commun. 2024 Oct 17;15:8967. doi: 10.1038/s41467-024-53379-y (PMC11487085; doi:10.1038/s41467-024-53379-y)
Supplement: Supplementary file 1 — Supplementary Information [file 41467_2024_53379_MOESM1_ESM.pdf]

Supplementary Materials for

Gustatory Interface for Operative Assessment and Taste Decoding in Patients with Tongue Cancer

Xiner Wang[1,3]†, Guo Bai[4]†, Jizhi Liang[1,3]†, Qianyang Xie[4]†, Zhaohan Chen[7], Erda Zhou[1,3], Meng Li[2,3], Xiaoling Wei[2,3], Liuyang Sun[1,3], Zhiyuan Zhang[4], Chi Yang[4*], Tiger H. Tao[1,2,3,5,6,7,8,9*] and Zhitao Zhou[2,3*]

[1]2020 X-Lab, Shanghai Institute of Microsystem and Information Technology, Chinese Academy of Sciences, Shanghai 200050, China

[2]State Key Laboratory of Transducer Technology, Shanghai Institute of Microsystem and Information Technology, Chinese Academy of Sciences, Shanghai 200050, China

[3]School of Graduate Study, University of Chinese Academy of Sciences, Beijing 100049, China

[4]Department of Oral Surgery, Shanghai Ninth People's Hospital, Shanghai Jiao Tong University School of Medicine; College of Stomatology, Shanghai Jiao Tong University; National Center for Stomatology; National Clinical Research Center for Oral Diseases; Shanghai Key Laboratory of Stomatology; Shanghai Research Institute of Stomatology; Research Unit of Oral and Maxillofacial Regenerative Medicine, Chinese Academy of Medical Sciences, Shanghai 200011, China

[5]Center of Materials Science and Optoelectronics Engineering, University of Chinese Academy of Sciences, Beijing 100049, China

[6]Center for Excellence in Brain Science and Intelligence Technology, Chinese Academy of Sciences, Shanghai 200031, China

[7]Neuroxess Co., Ltd., Shanghai 200023, China

[8]Guangdong Institute of Intelligence Science and Technology, Hengqin, Zhuhai, Guangdong 519031, China

[9]Tianqiao and Chrissy Chen Institute for Translational Research, Shanghai, China.

*Corresponding author. Email: yangchi63@hotmail.com; tiger@mail.sim.ac.cn; ztzhou@mail.sim.ac.cn

†These authors contributed equally to this work.

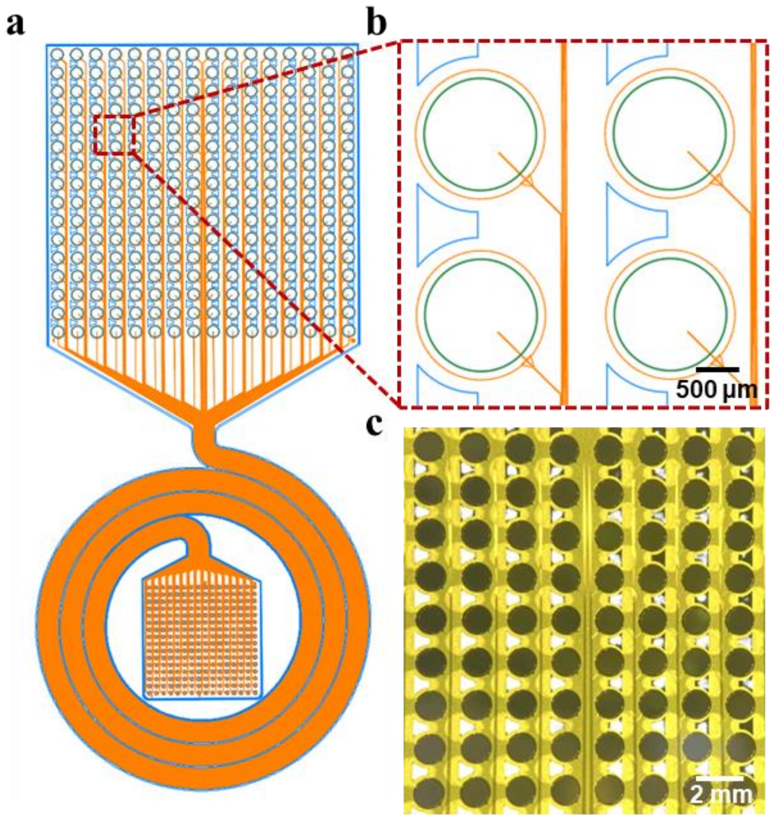

Supplementary Figure 1. Design of the tongue electrodes. a, The overall layout of the 256-channel tongue electrodes. **b,** Enlarged diagram depicting four recording sites surrounded by mesh structures, with the openings of each recording site are 1.3 mm in diameter. **c,** Microscope image of the tongue electrodes.

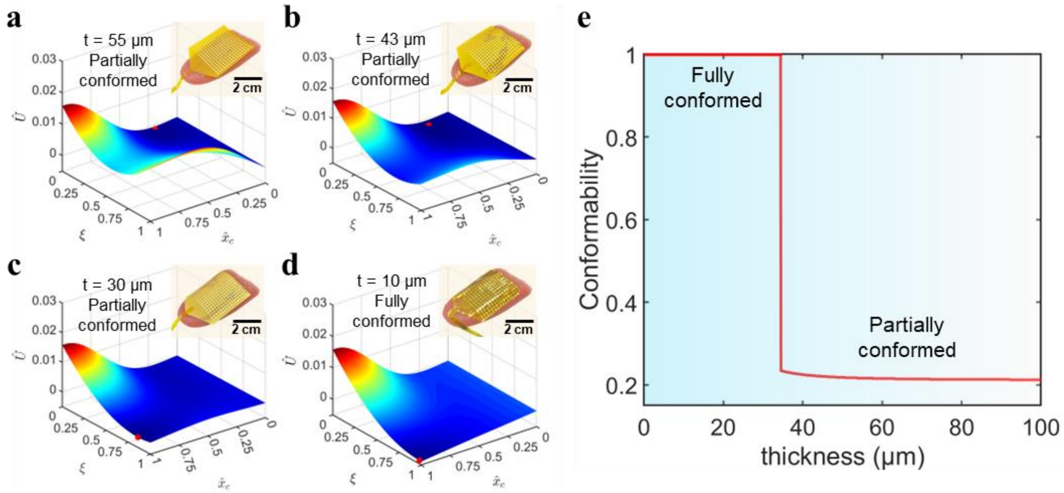

Supplementary Figure 2. Adhesion simulation. Simulation of the normalized total energy of the system where flexible electrodes vary in thickness: 55 µm **(a)**, 43 µm **(b)**, 30 µm **(c)** and 10 µm **(d)**. Red dots highlight global minima. Insets display the photographs of corresponding tongue electrodes placing on a tongue model. **e,** Relationship between degree of conformability and device thickness.

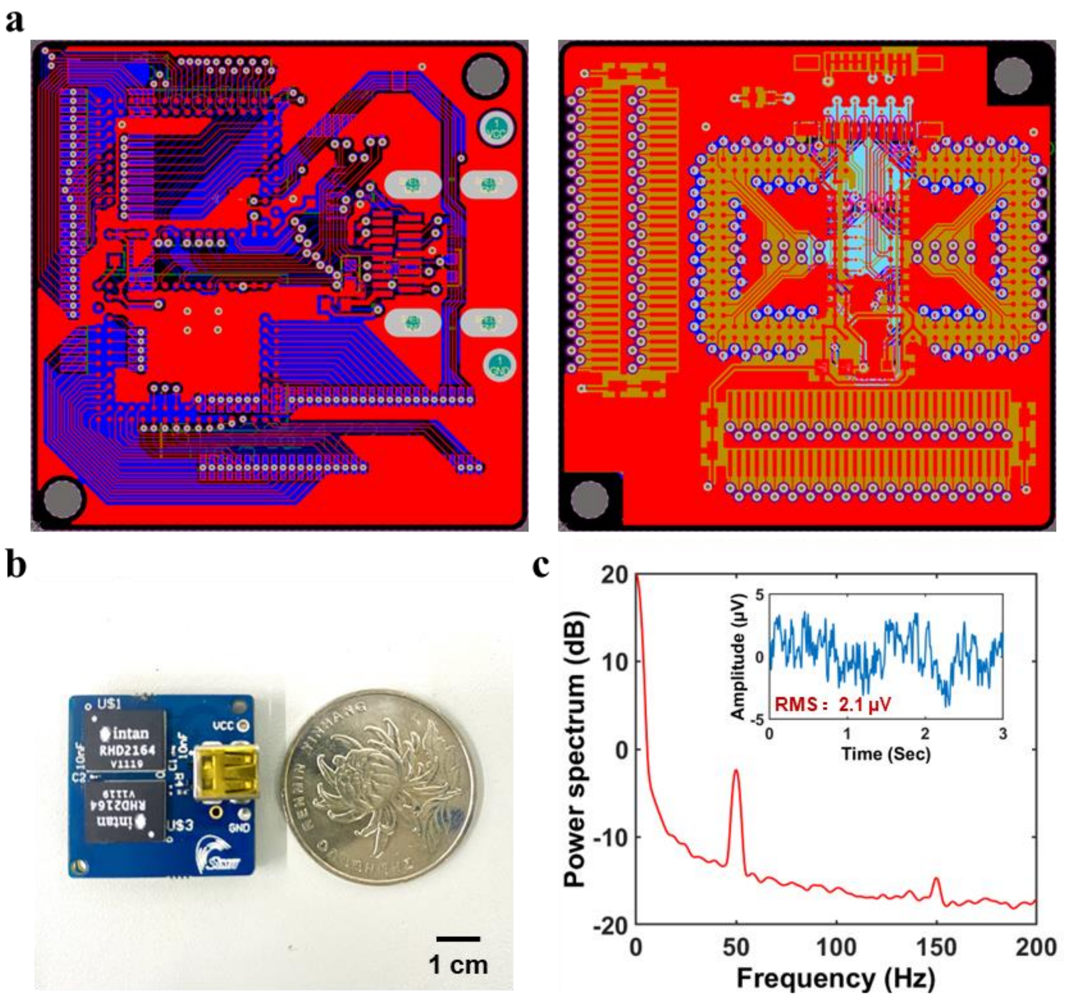

Supplementary Figure 3. Design of the customized headstage. a, Layout design of the custom two-layer stacked PCB for signal recording. Such unit can simultaneously collect, amplify, and digitalize 256-channel signals through four 64-pin Molex interfaces. **b,** Image of the custom headstage, nearly comparable in size to a coin. **c,** Spectrum of the signal recorded from the tongue electrodes immersed in 1× PBS solution using our customized headstage, demonstrating relatively low-noise performance. Inset shows the time traces and the root mean square (RMS) value.

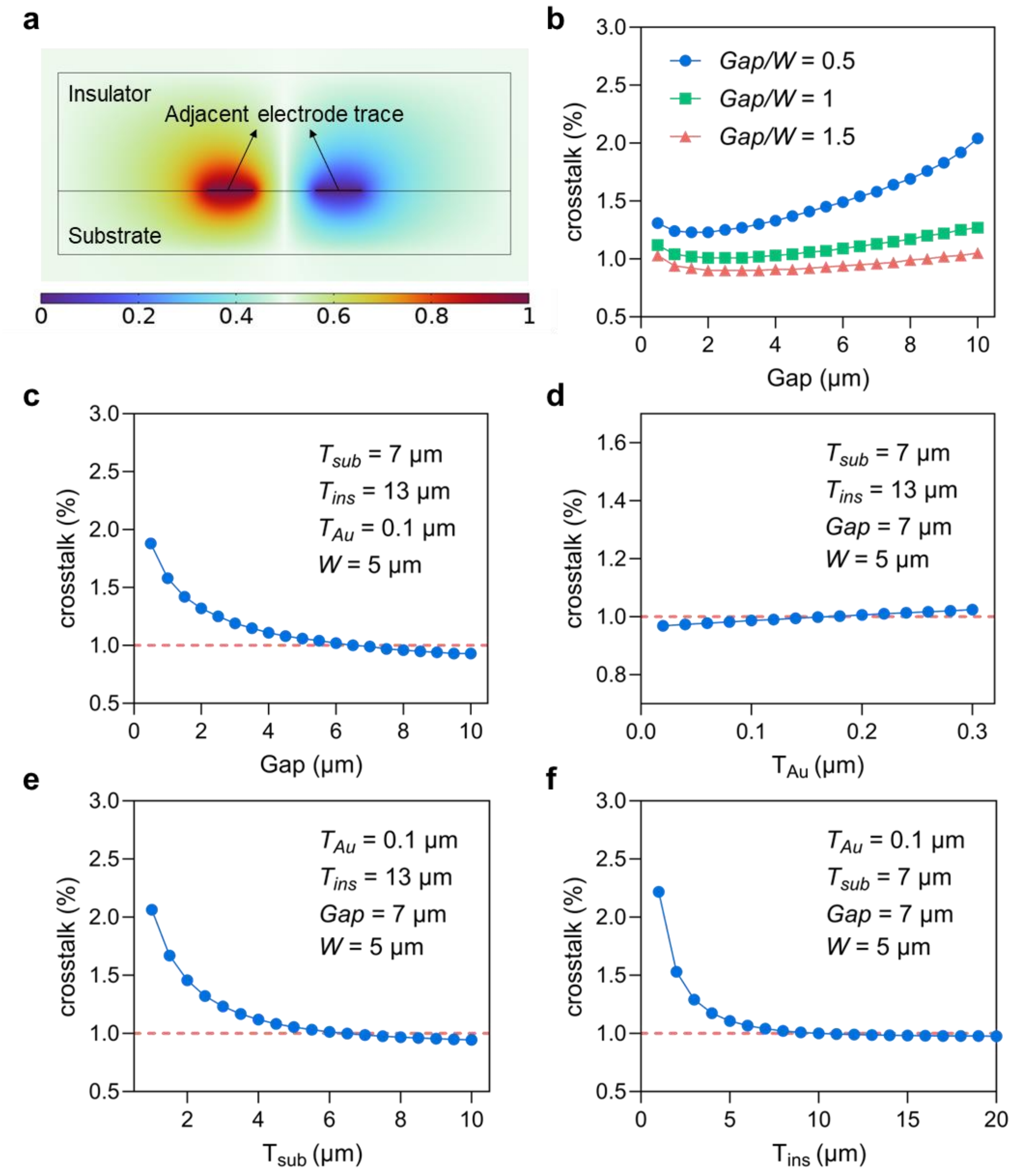

Supplementary Figure 4. Simulation of the crosstalk. a, Simulation model with electrostatic potential mapping. **b,** Simulated crosstalk as a function of the gap between adjacent electrode traces in 1x PBS solution with different ratios of the gap to the width of the trace. Simulated crosstalk as a function of the trace gap **(c)**, the thickness of Au trace **(d)**, the thickness of the substrate **(e)**, and the thickness of the insulator **(f)**.

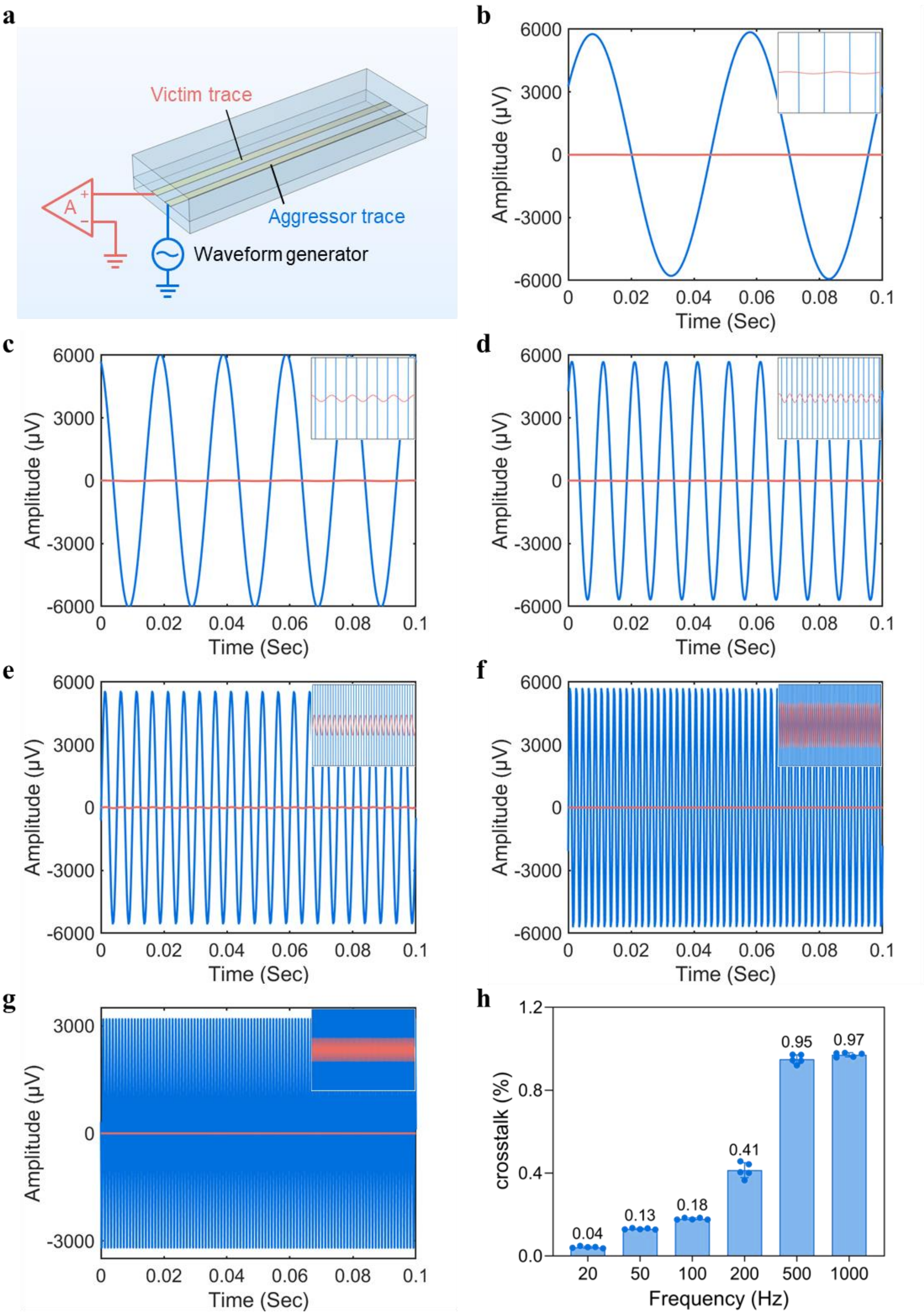

Supplementary Figure 5. *In vitro* evaluation of crosstalk between neighboring traces. a, The *in vitro* setup of crosstalk evaluation. Representative waveforms of the input sinusoidal signals (blue) to the aggressor trace with the frequency of 20 Hz **(b)**, 50 Hz **(c)**, 100 Hz **(d)**, 200 Hz **(e),** 500 Hz **(f),** 1000 Hz **(g)** and the recorded signals (pink) acquired from the victim trace. Zoom-in views are enlarged waveforms detected from the victim trace. **h,** Bar plots illustrating the crosstalk under different frequencies. Data are presented as mean values +/- SD.

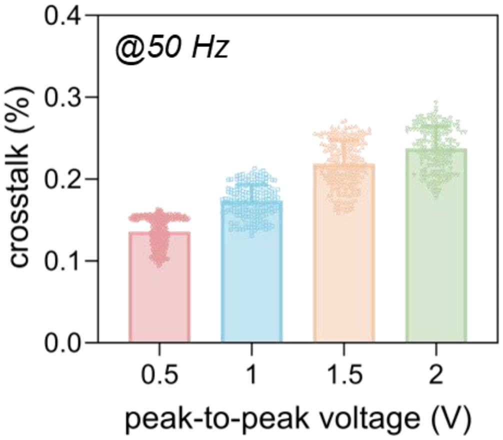

Supplementary Figure 6. Bar plots illustrating the crosstalk at various test signals with different peak-to-peak voltages under the frequency of 50 Hz. Data are presented as mean values +/- SD.

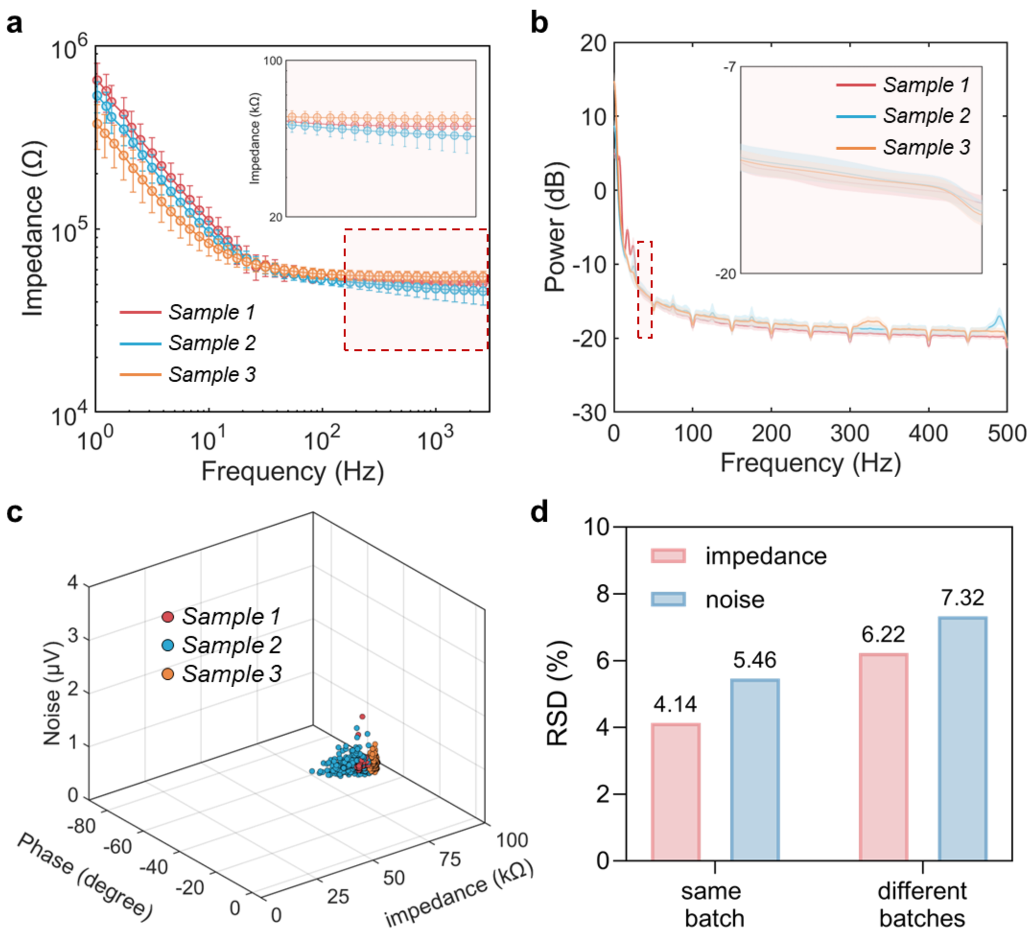

Supplementary Figure 7. Uniformity and reproducibility of electrical performance and signal measurements between electrodes prepared in the same and different batches. a, EIS plots for all recording sites from the electrode sample 1, 2, and 3 in 1x PBS buffer. Data are presented as mean values +/- SD. **b,** PSD for all recording sites from the electrode sample 1, 2, and 3 in 1x PBS buffer. Data are presented as mean values +/- SD. **c,** The comparisons of impedance (at 1 kHz), phase (at 1 kHz), and RMS noise of the signals for different electrodes. **d,** RSD between the electrodes in the same batch and from different batches.

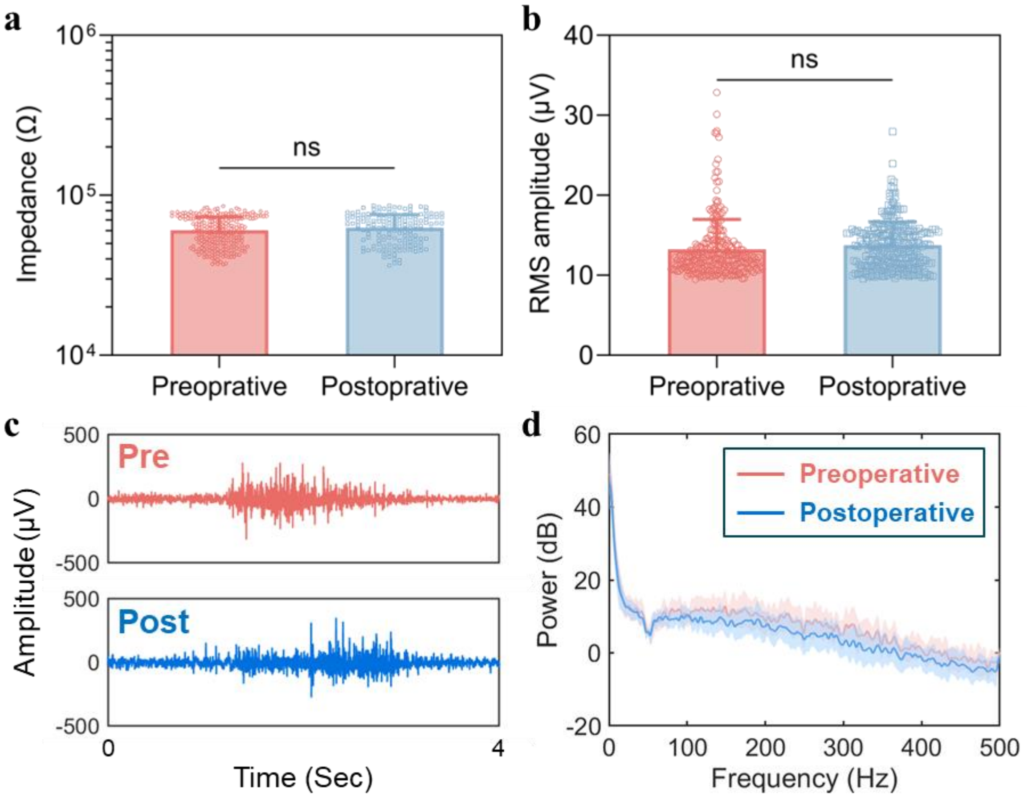

Supplementary Figure 8. Uniformity of signals acquired from the same patient before and after the surgery. a, Impedance of the electrodes attached to the tongue surface of the patient preoperative and postoperative. Data are presented as mean values +/- SD. $p = 0.0534$. (ns, $p > 0.05$, *$p < 0.05$, **$p < 0.01$, ***$p < 0.001$, two-sided unpaired t test) **b,** The RMS values of the resting-state signal amplitude on the patient preoperative and postoperative. Data are presented as mean values +/- SD. $p = 0.1001$. (ns, $p > 0.05$, *$p < 0.05$, **$p < 0.01$, ***$p < 0.001$, two-sided unpaired t test) **c,** Time traces of the representative TE signals evoked by taste stimulation acquired from the natural tongue preoperative and postoperative. **d,** Power spectral densities of the signals in **c**. Data are presented as mean values +/- SD.

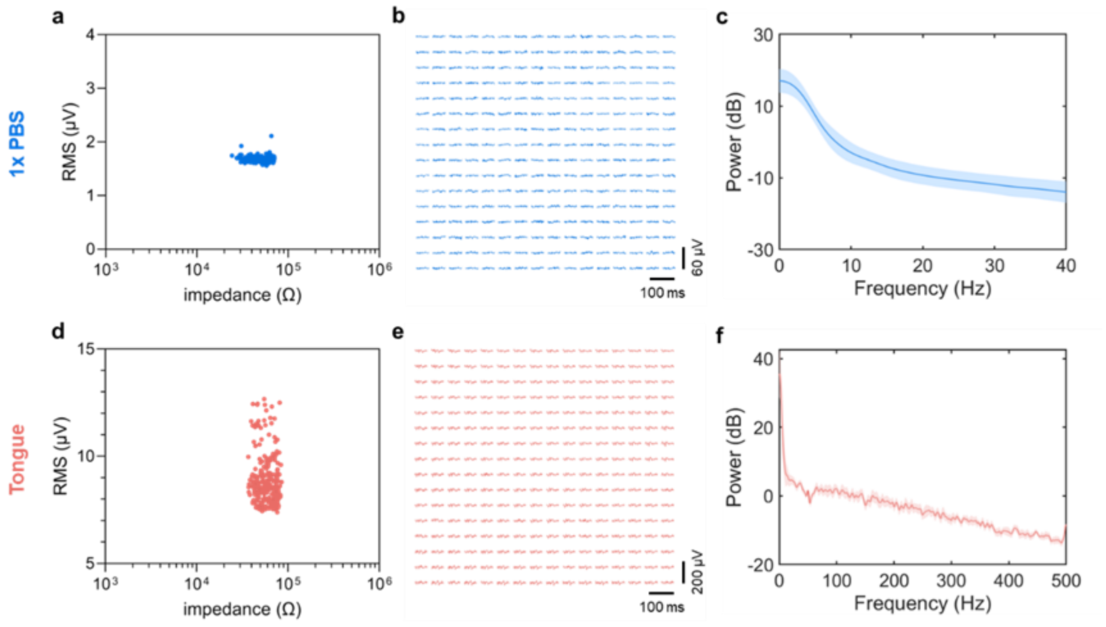

Supplementary Figure 9. Uniformity of the impedances and baseline signals for the tongue electrodes. RMS levels of baseline signals and impedances were estimated for all 256 electrode channels in 1x PBS **(a)** and on tongue surface **(d)**. Sensing baselines for each recording site of the tongue electrodes in 1x PBS **(b)** and on tongue surface **(e)**. Power spectral densities of sensing baselines in 1x PBS **(c)** and on tongue surface **(f)**. Data are presented as mean values +/- SD.

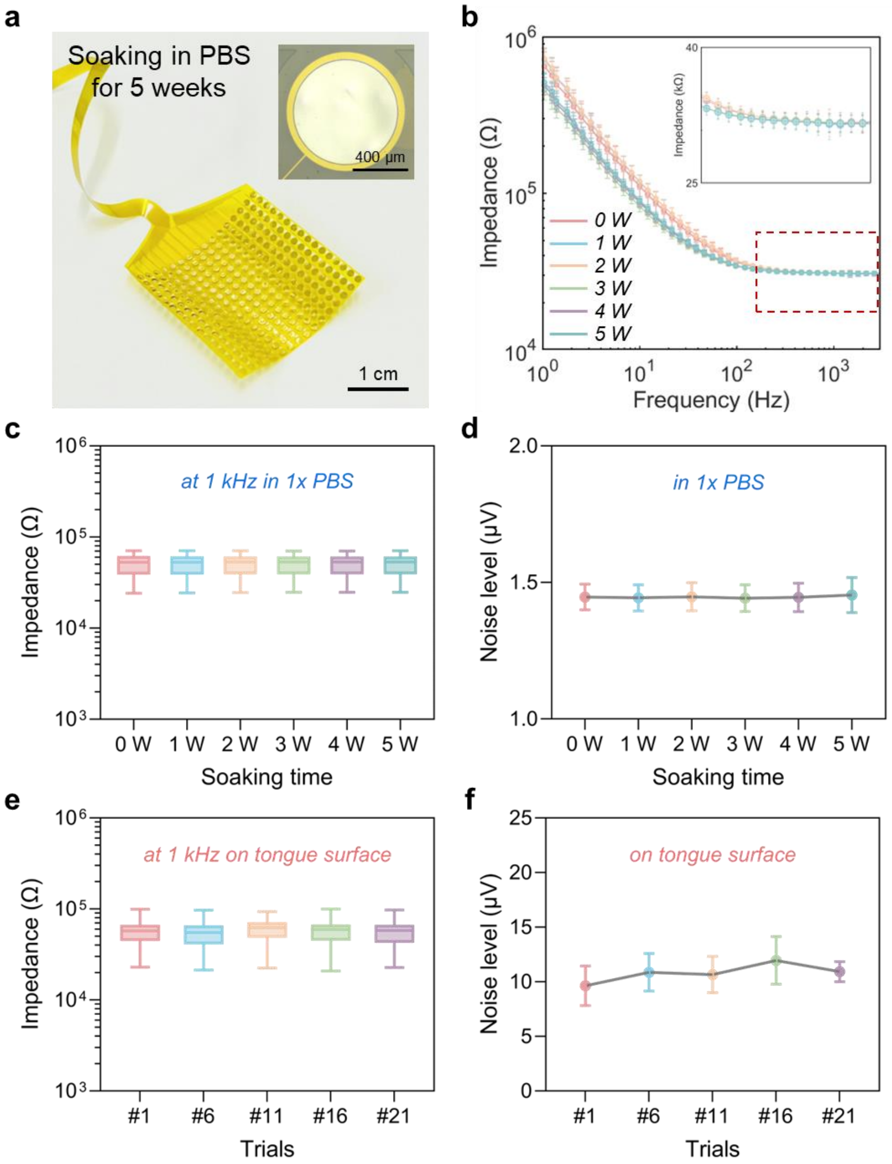

Supplementary Figure 10. Performance stability of the tongue electrodes. a, Photograph of the tongue electrode after 5 weeks of immersion in 1x PBS. Inset: zoomed-in microscope image of the metal contact without detachment and corrugation. **b,** Electrochemical impedance characterization of the tongue electrode soaked in 1x PBS solution for 5 weeks. Data are presented as mean values +/- SD. **c,** Impedance stability of the tongue electrode at 1 kHz over time when immersed in 1x PBS solution. **d,** Characterization of the noise level showing good stability in the ability to acquire signals of the electrode incubated in 1x PBS solution for 5 weeks. **e,** Impedance changes of the electrode on tongue surface over multiple trials. **f,** Relatively stable and low noise

level across multiple trials confirmed the stability for electrophysiological recording. Data in **d** and **f** are presented as mean values +/- SD. Box plots in **c** and **e** display the data median (centre line), upper and lower quartiles (bounds of box), 1.5 times the interquartile range (whiskers) and outlier values beyond this range (circles).

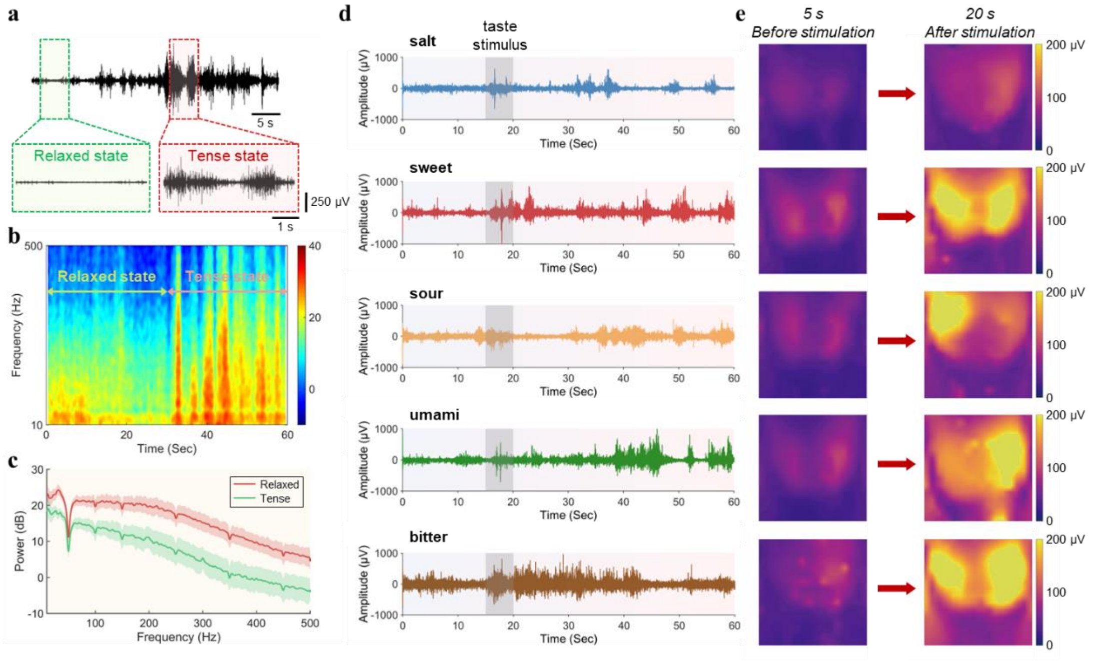

Supplementary Figure 11. Tongue electrical signals acquired from normal subjects. **a,** Filtered (10-500 Hz) time traces of TE signals during the relaxed and tense state of the tongue. **b,** Corresponding time-frequency spectrogram of TE signals in **(a)** highlighting an increase in power in the frequency band below 500 Hz. **c,** Power spectral density of TE signals during relaxed and tense states. Data are presented as mean values +/- SD. **d,** 60 seconds of example filtered (10-500 Hz) responses recorded from a representative channel of the flexible tongue electrodes evoked by five taste stimuli respectively. The shaded grey area indicates the epoch when the taste stimuli were presented. **e,** Dynamic change of spatial power maps of TE signals. The first column denotes the five seconds before the taste stimuli, and the second represents the twenty seconds after the stimuli. Power is calculated every 1 s epoch.

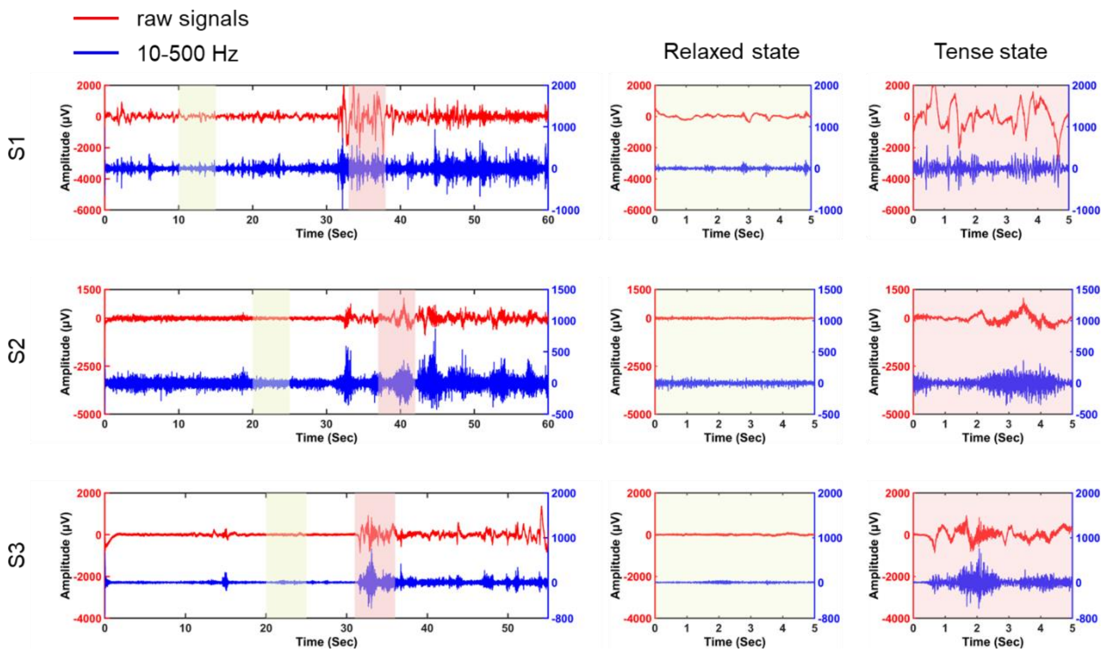

Supplementary Figure 12. TE signals acquired from 3 normal subjects (S1, S2, and S3) without taste stimulation. The raw and filtered data are indicated by red and blue lines, demonstrating the removal of excess noise through filtering. A zoomed-in view of five seconds of signals in both relaxed and tense states reveal an evident difference.

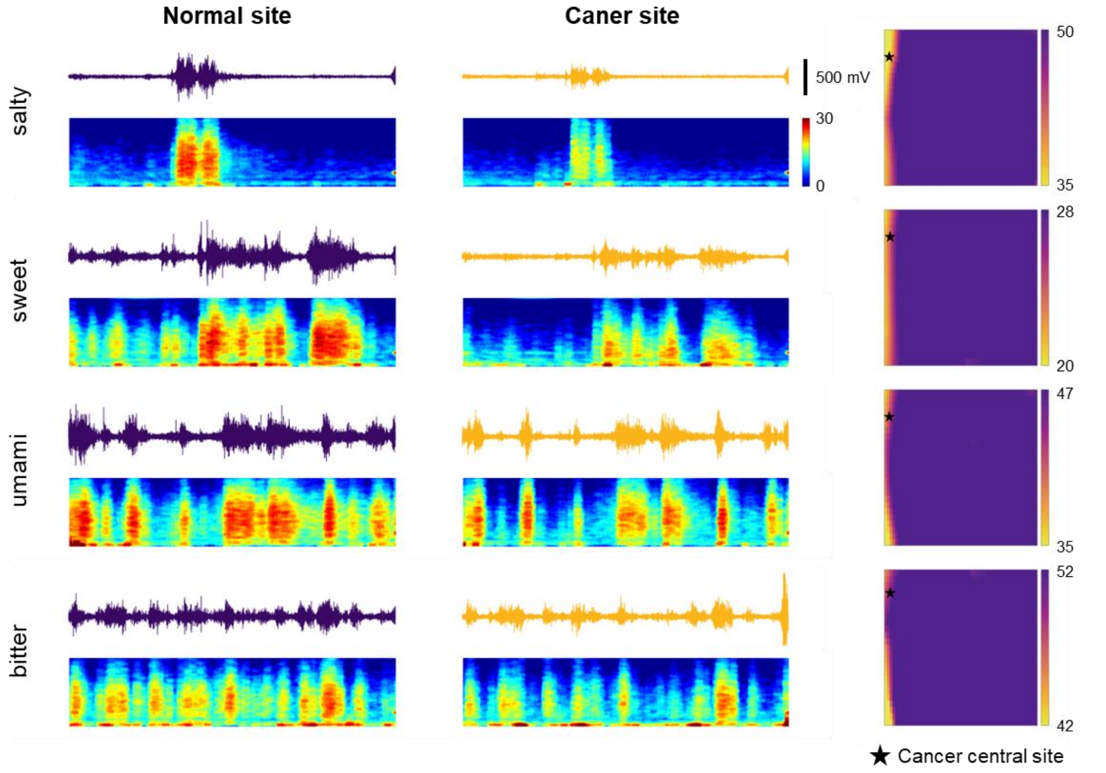

Supplementary Figure 13. Preoperative tongue cancer localization under salty, sweet, umami, and bitter stimulations. Left is the representative TE signals and time-frequency spectra from the normal site and the cancer site. Right is the power heatmaps of 256 channels of TE signals, consistent with the findings in Fig. 2. The cancer central site is highlighted by the dark star.

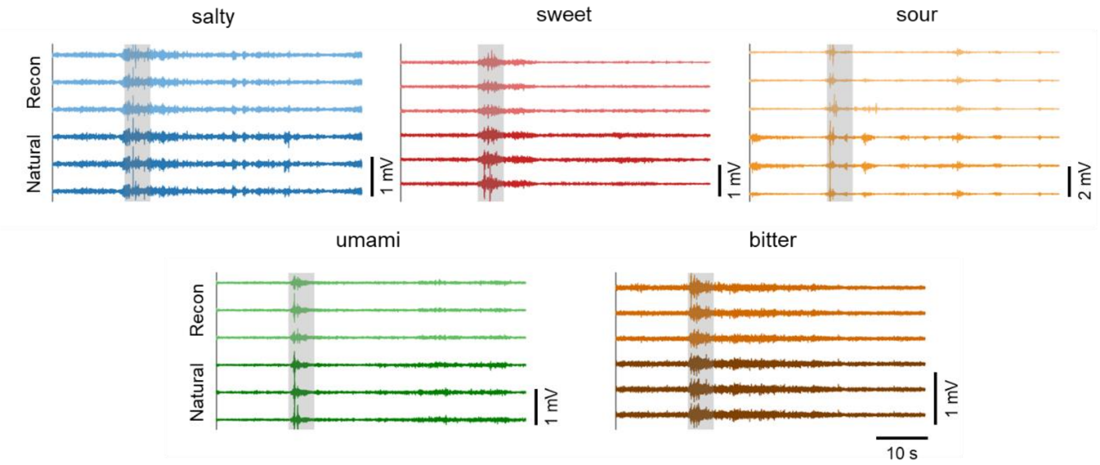

Supplementary Figure 14. Multi-channel TE signals acquired from the reconstructed and natural tongue under taste stimulations. Each plot shows 3-channel TE signals of the reconstructed tongue (top) and 3-channel TE signals of the natural tongue (bottom) evoked by different tastes. The taste-induced TE activities on the natural part are relatively stronger than those on the reconstructed one.

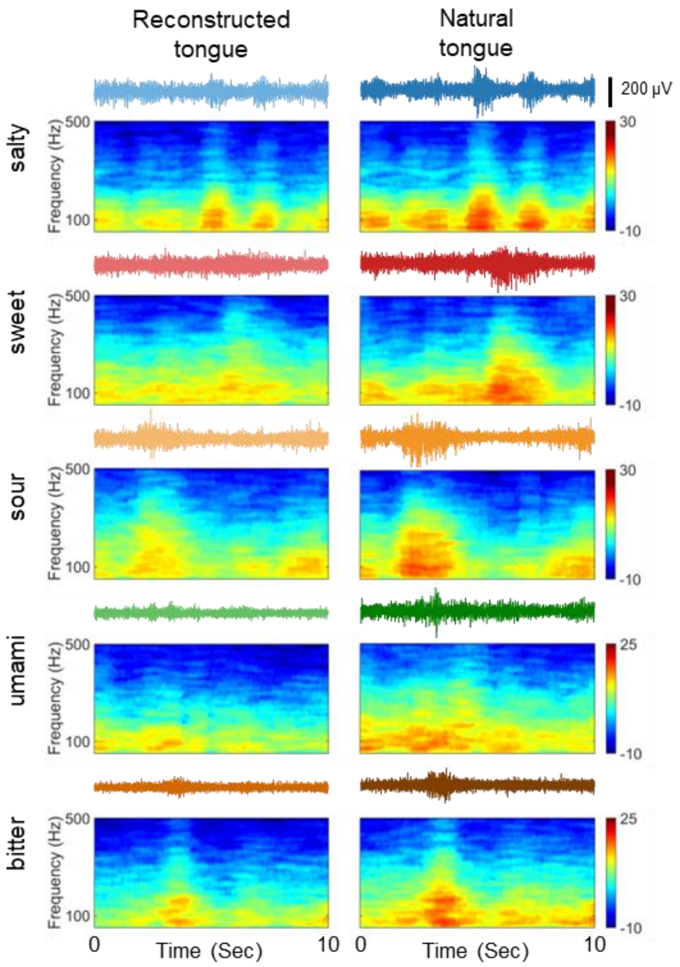

Supplementary Figure 15. Representative TE signals acquired from reconstructed and natural tongue under taste stimulations. The top row of each taste stimulation shows the time-domain waveforms of specific TE signals, while the bottom row displays the corresponding time-frequency spectra.

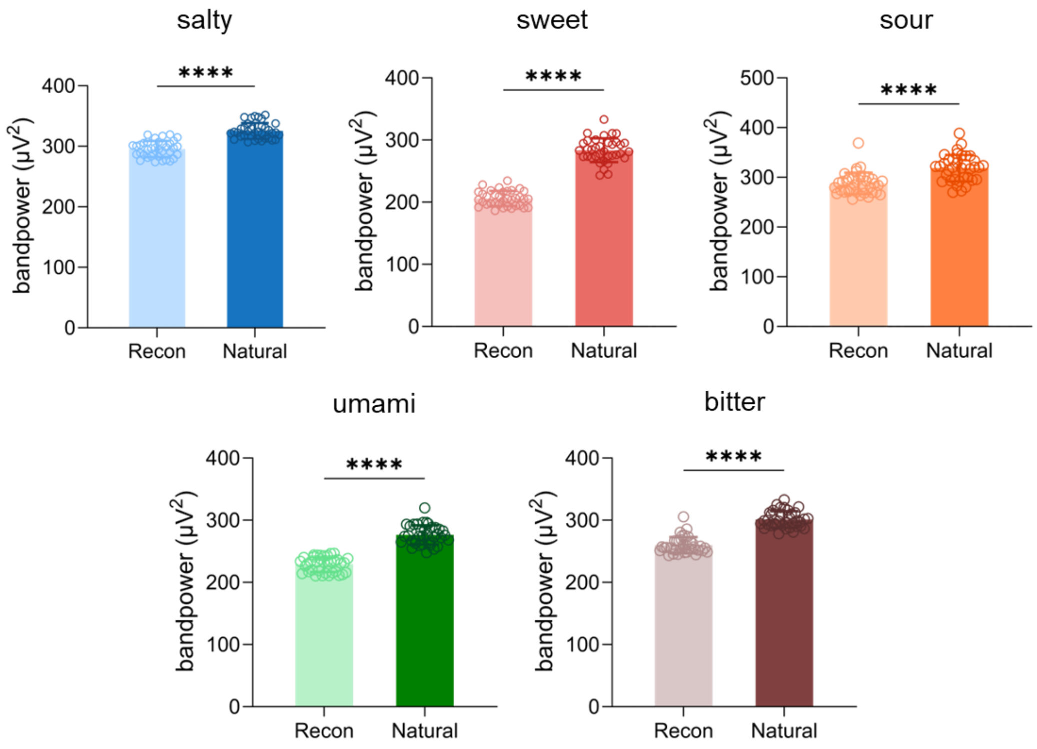

Supplementary Figure 16. Power distribution of TE signals from the reconstructed and natural tongue. Each paired bar depicts the mean power of the TE signals recorded from the reconstructed tongue (left, Recon) and the natural tongue (right, Natural). A significant difference is observed between these two parts of the tongue under each of the five taste stimulations. Data are presented as mean values +/- SD. $p = 2.53\times10^{-14}$, salty; $p = 1.90\times10^{-31}$, sweet; $p = 7.99\times10^{-7}$, sour; $p = 9.63\times10^{-23}$, umami; $p = 7.20\times10^{-21}$, bitter. (*$p < 0.05$, **$p < 0.01$, ***$p < 0.001$, ****$p < 0.0001$, two-sided unpaired *t* test)

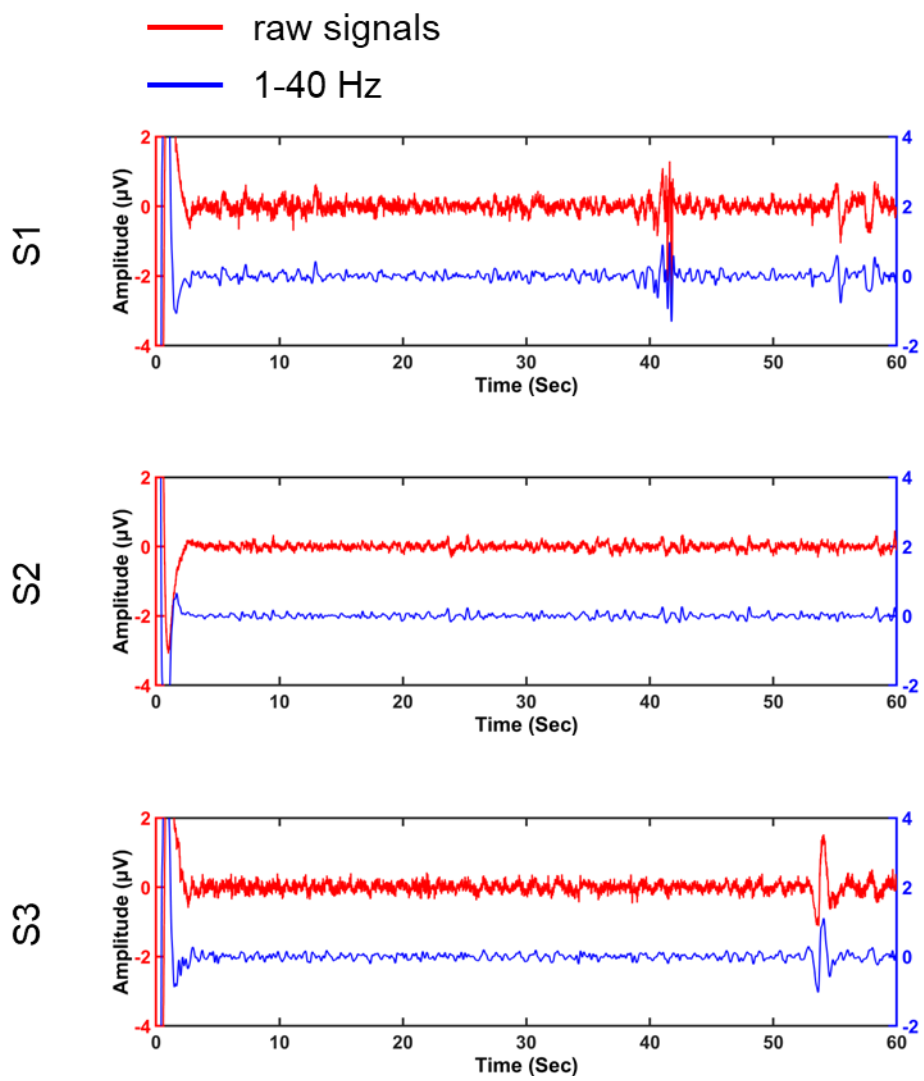

Supplementary Figure 17. EEG signals acquired from 3 normal subjects (S1, S2, and S3) without taste stimulation. The raw and filtered data are represented by red and blue lines, respectively.

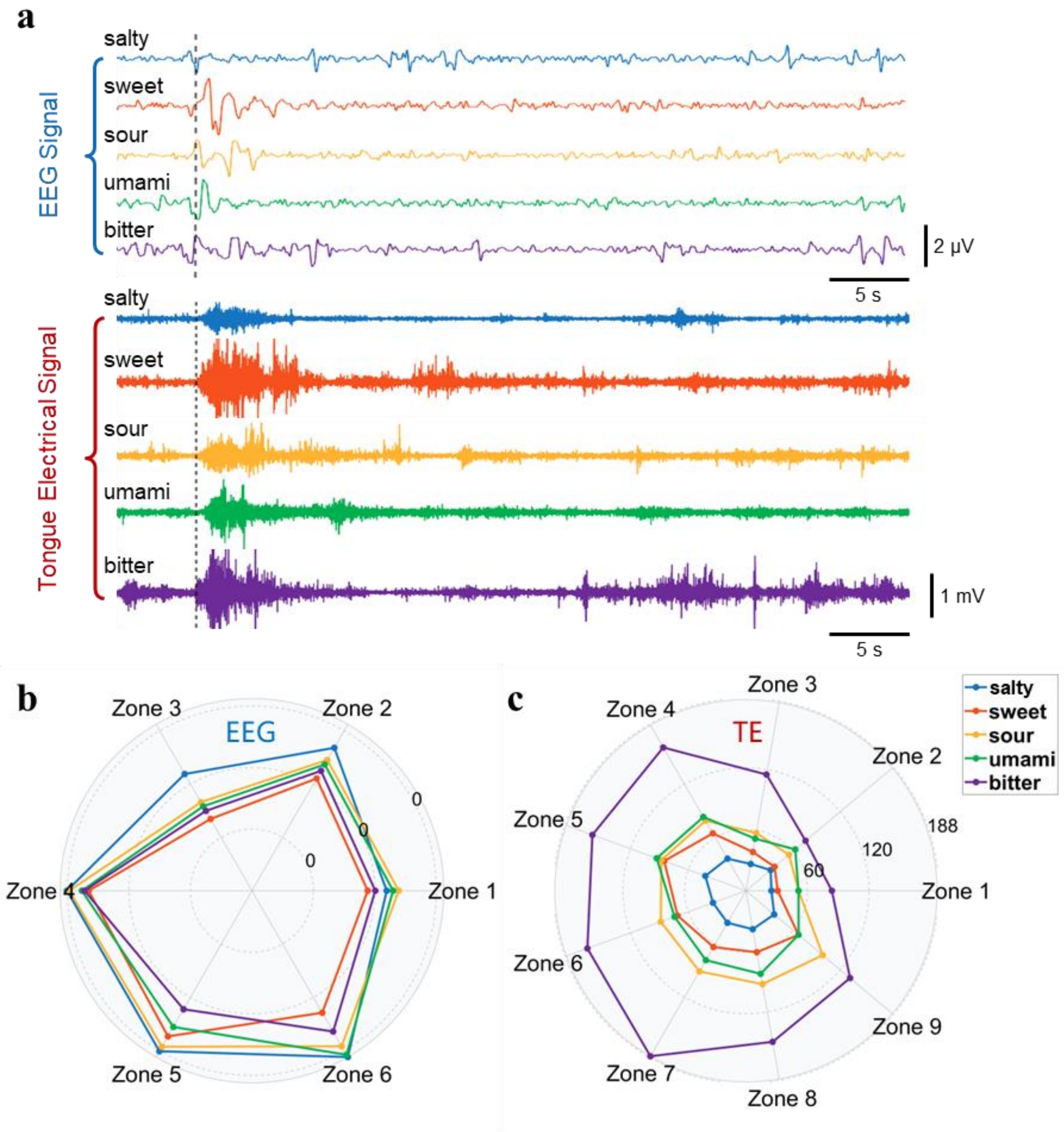

Supplementary Figure 18. TE and EEG signals acquired from S3 under taste stimulations. a, Representative EEG and tongue electrical signals simultaneously acquired from S3 in response to five taste stimulations. Zone-distribution radar maps of the intensity of EEG signals **(b)** and TE signals **(c)** triggered by five taste stimulations applied on the tongue. Five taste stimulations induce EEG signals with minor discrepancies in all zones, with salty eliciting the largest signal intensity. The order of the strength in the obtained TE signals generally maintains a consistent trend across different zones.

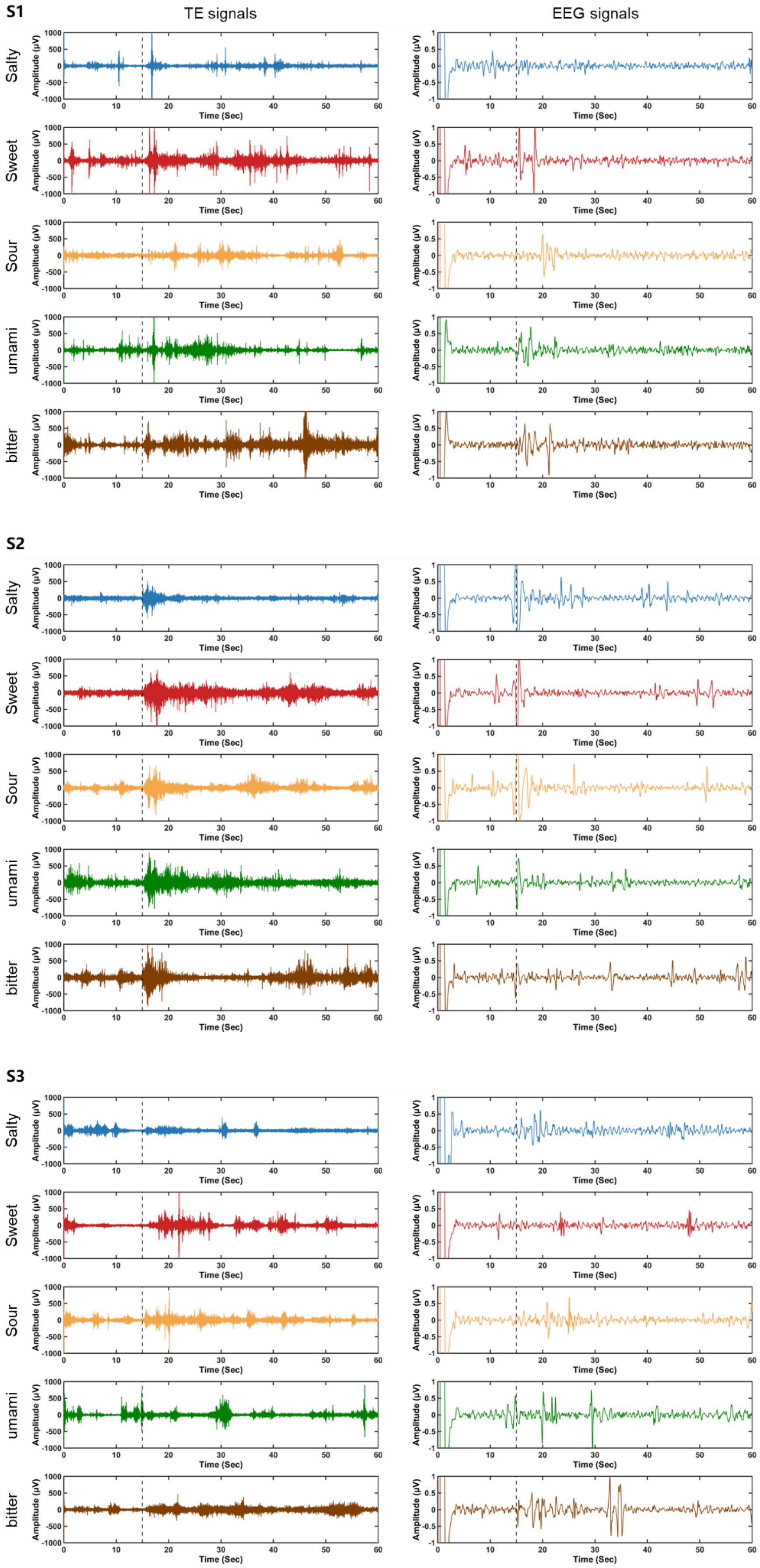

S1
TE signals
EEG signals
Salty
Sweet
Sour
umami
bitter
S2
S3
Amplitude (μV)
Time (Sec)

Supplementary Figure 19. TE and EEG signals acquired from 3 normal subjects (S1, S2, and S3) under five types of taste stimulations. The TE signals are filtered to 10-500 Hz while EEG signals are filtered with the 1-40 Hz band. The dotted line indicates the onset of the taste stimulation.

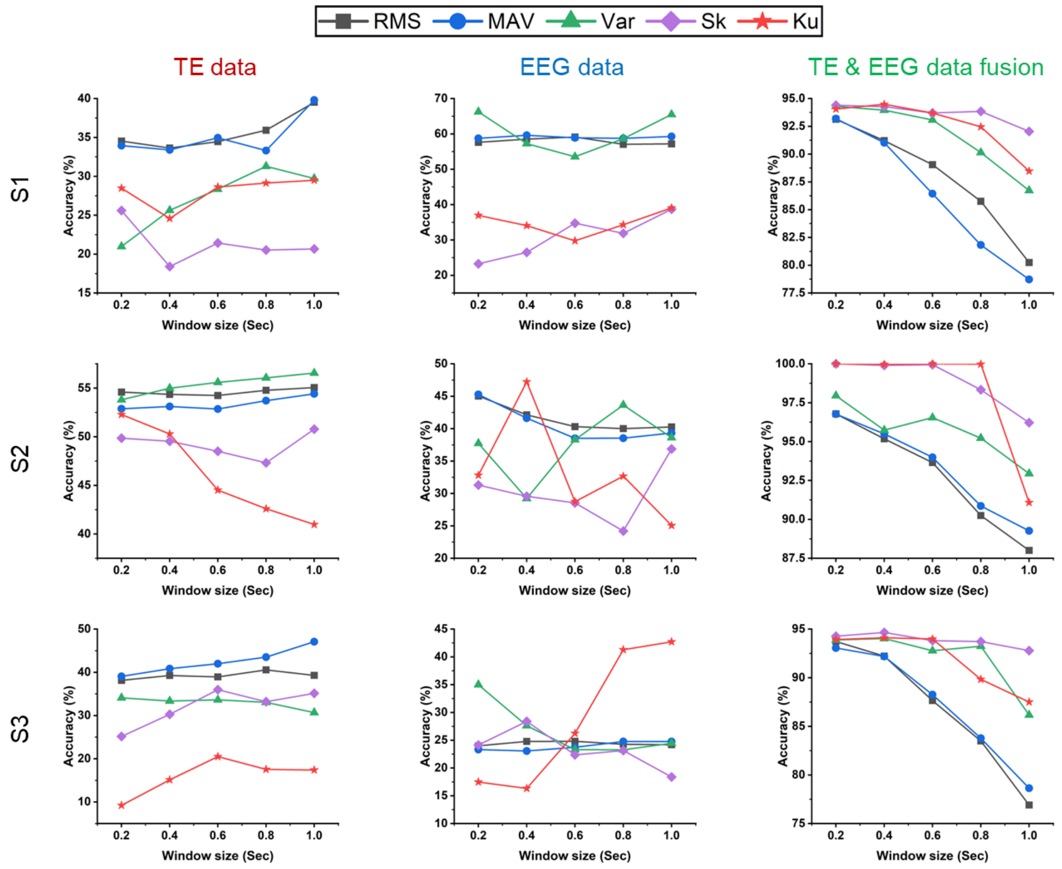

Supplementary Figure 20. Classification accuracy of different time-domain features on individual normal subjects. Five time-domain features, namely RMS, MAV, Var, Sk and Ku, are extracted from both TE and EEG signals. Each row illustrates the classifier accuracy using TE, EEG and fused features for a specific normal subject.

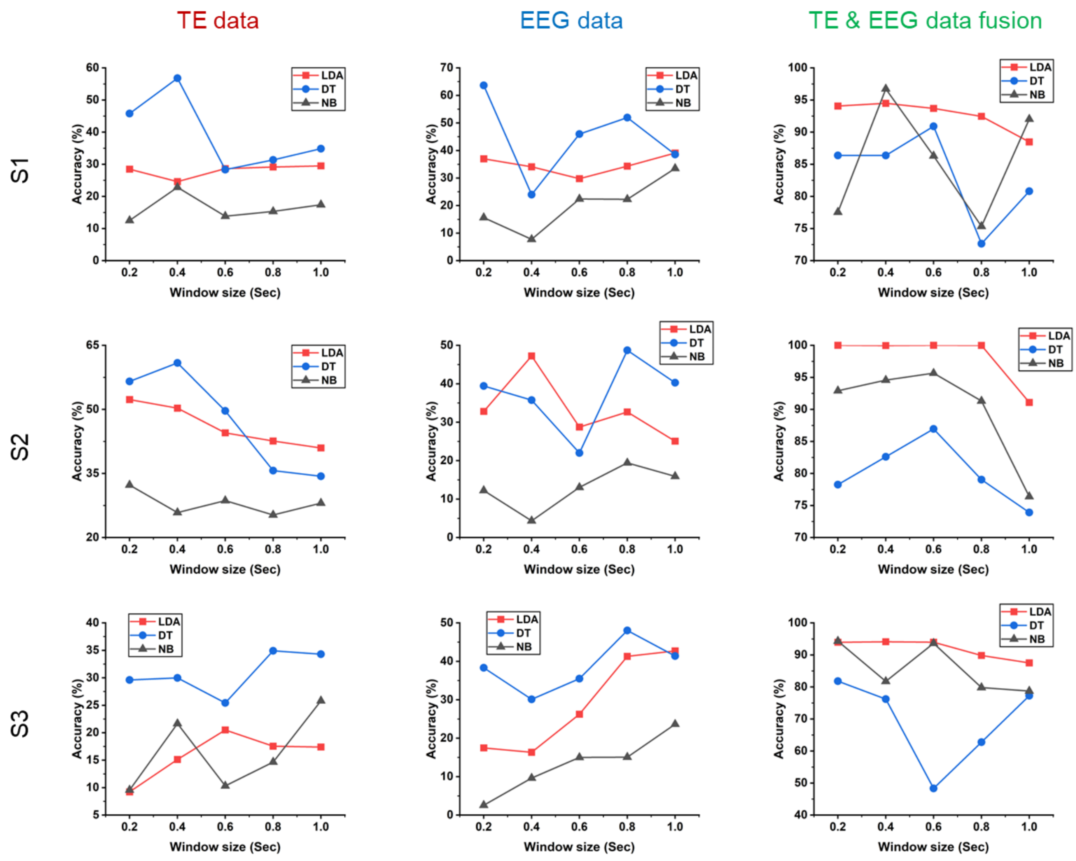

Supplementary Figure 21. Classification accuracy of different classifiers on individual normal subjects. Three widely-used machine learning classifiers - LDA, DT and NB are adopted for taste classification. Each row illustrates the classifier accuracy using TE, EEG and fused features for a specific normal subject. The LDA classifier exhibits superior performance compared to the other two classifiers when TE and EEG data are fused.

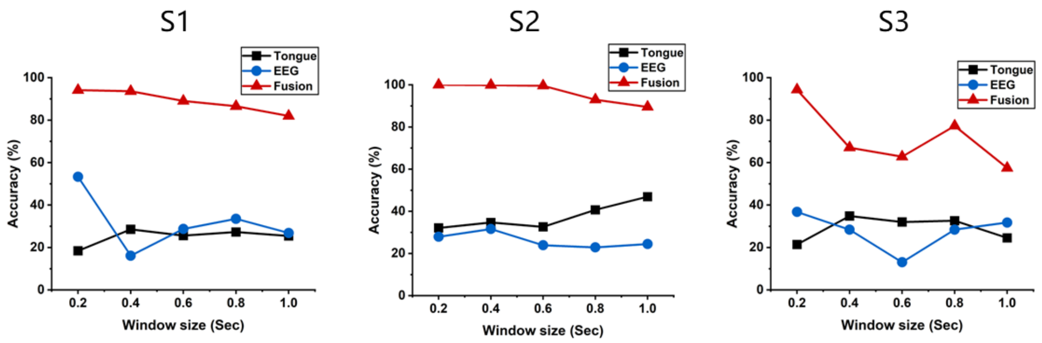

Supplementary Figure 22. Classification using single and fused modalities on individual normal subjects. Each plot shows the LDA classifier accuracy using Ku features extracted from TE, EEG and the fused data for each normal subject. The results highlight the effectiveness of the dual-modal fusion strategy in taste classification, with a sliding window of 0.2 seconds appearing relatively optimal.

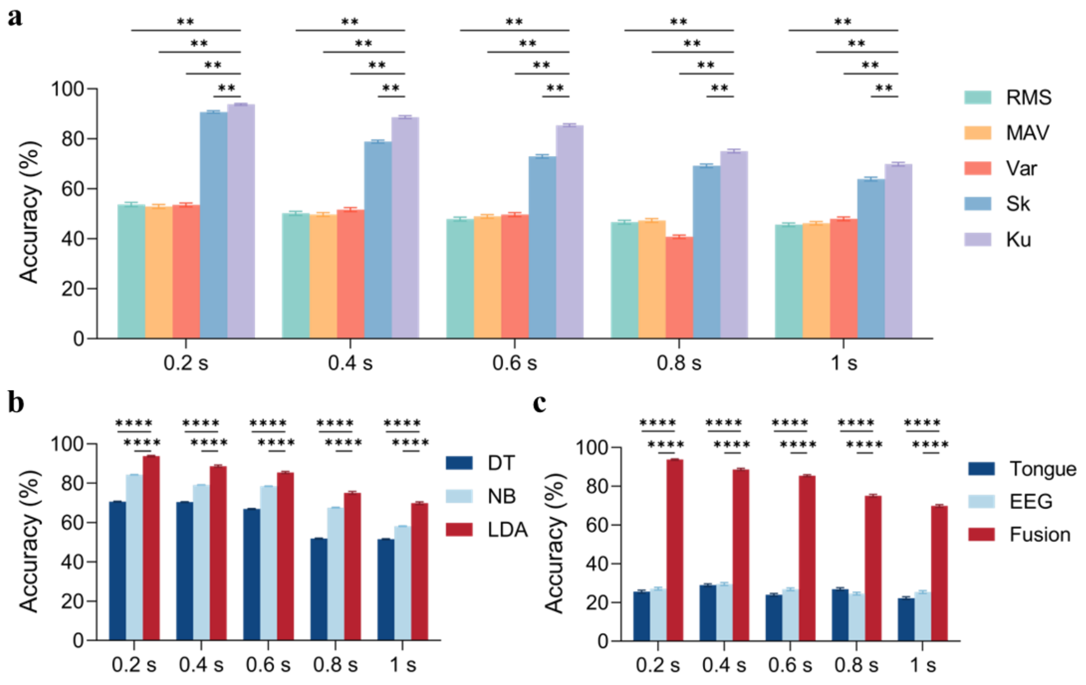

Supplementary Figure 23. Summary of the classification performance on different parameters. a, Bar plots illustrating the classification accuracy of five features. Data are presented as mean values +/- SEM. (**p* < 0.05, ***p* < 0.01, ****p* < 0.001, *****p* < 0.0001; compared to the accuracy of feature Ku, two-way ANOVA). **b,** Bar plots illustrating the classification accuracy of three classifiers. Data are presented as mean values +/- SEM. (**p* < 0.05, ***p* < 0.01, ****p* < 0.001, *****p* < 0.0001; compared to the accuracy of LDA, two-way ANOVA). **c,** Bar plots illustrating the classification accuracy using only tongue or EEG data and fused data. Data are presented as mean values +/- SEM. (**p* < 0.05, ***p* < 0.01, ****p* < 0.001, *****p* < 0.0001; compared to the accuracy using fused data, two-way ANOVA). Considering the results of comparisons, the feature of Ku and the LDA classifier were selected as the final classifier parameters. The relatively optimal sliding window size (i.e., 0.2 s) was empirically determined, striking a balance between accuracy and computational consumption. Source data are provided as a Source Data file.

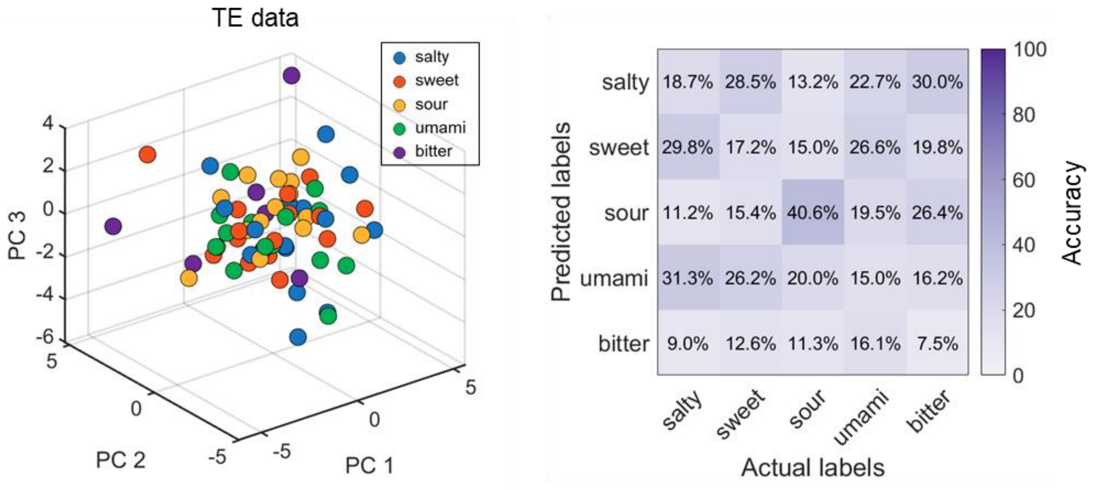

Supplementary Figure 24. Cross-subject classification using TE data. The plot (left) shows the feature space of the TE data acquired from all three normal subjects. The final classifier accuracy using the single modality of the TE features is demonstrated in the confusion matrix (right).

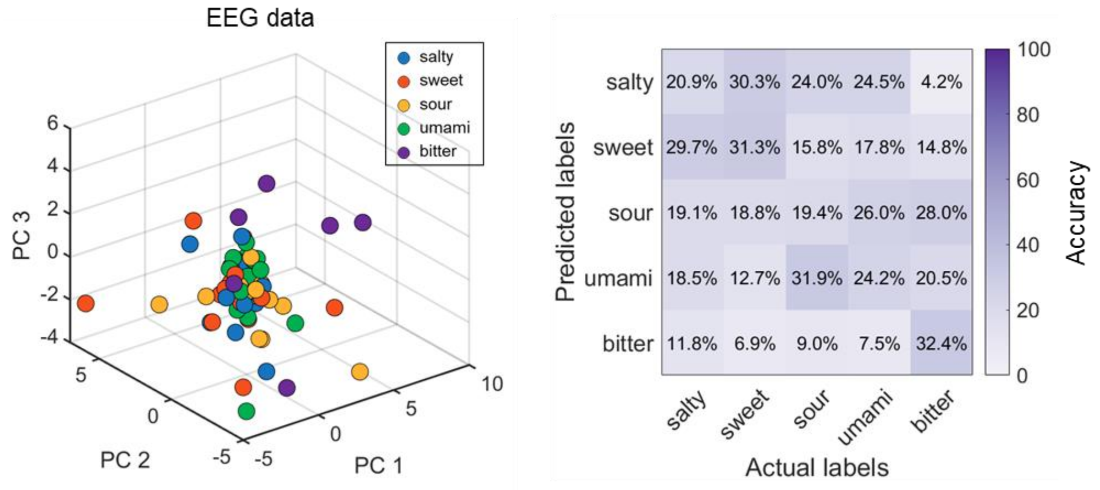

Supplementary Figure 25. Cross-subject classification using EEG data. The plot (left) shows the feature space of the EEG data acquired from all three normal subjects. The final classifier accuracy using the single modality of the EEG features is demonstrated in the confusion matrix (right).

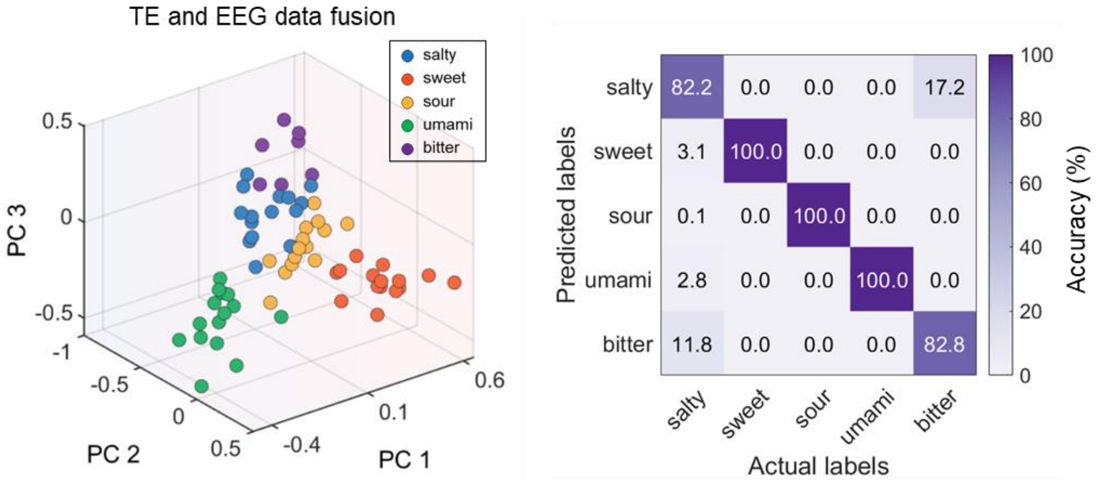

Supplementary Figure 26. Cross-subject classification using fused data of TE and EEG signals. The plot (left) shows the feature space of TE and EEG data fusion modality. The final classifier accuracy using fused TE and EEG data is demonstrated in the confusion matrix (right).

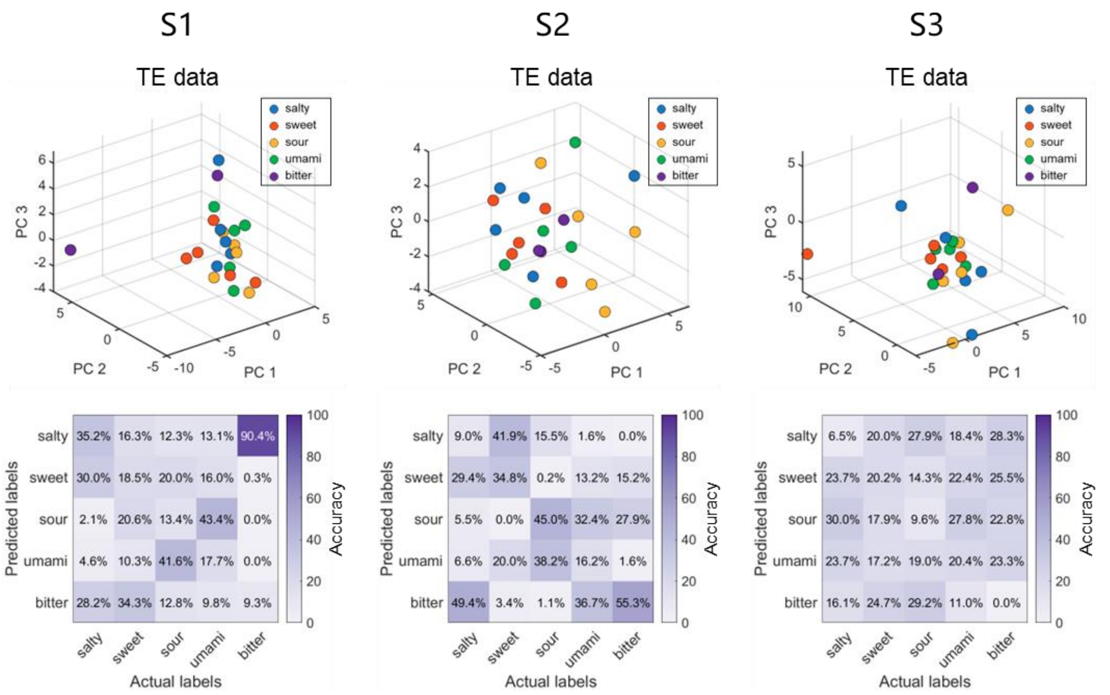

Supplementary Figure 27. Classification on individual subjects using TE data. The top row shows the feature space of the modality of TE data acquired from the three normal subjects, respectively. The classifier accuracy corresponding to each subject is shown in the confusion matrix of the bottom row.

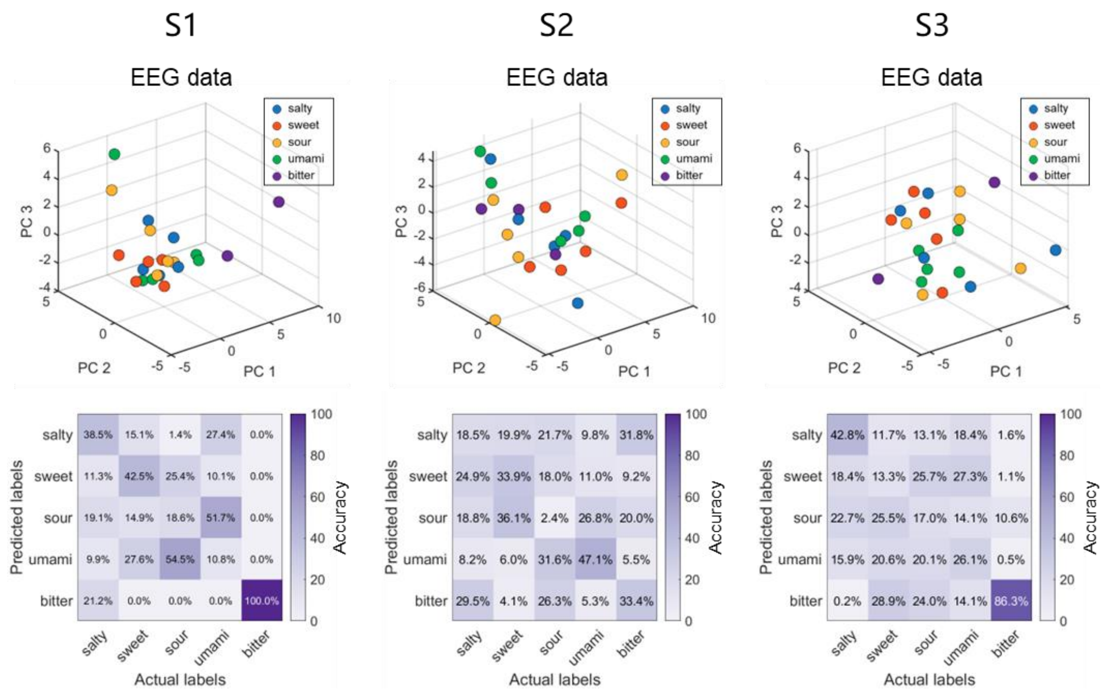

Supplementary Figure 28. Classification on individual subjects using EEG data. The top row shows the feature space of the modality of EEG data acquired from the three normal subjects, respectively. The classifier accuracy corresponding to each subject is shown in the confusion matrix of the bottom row.

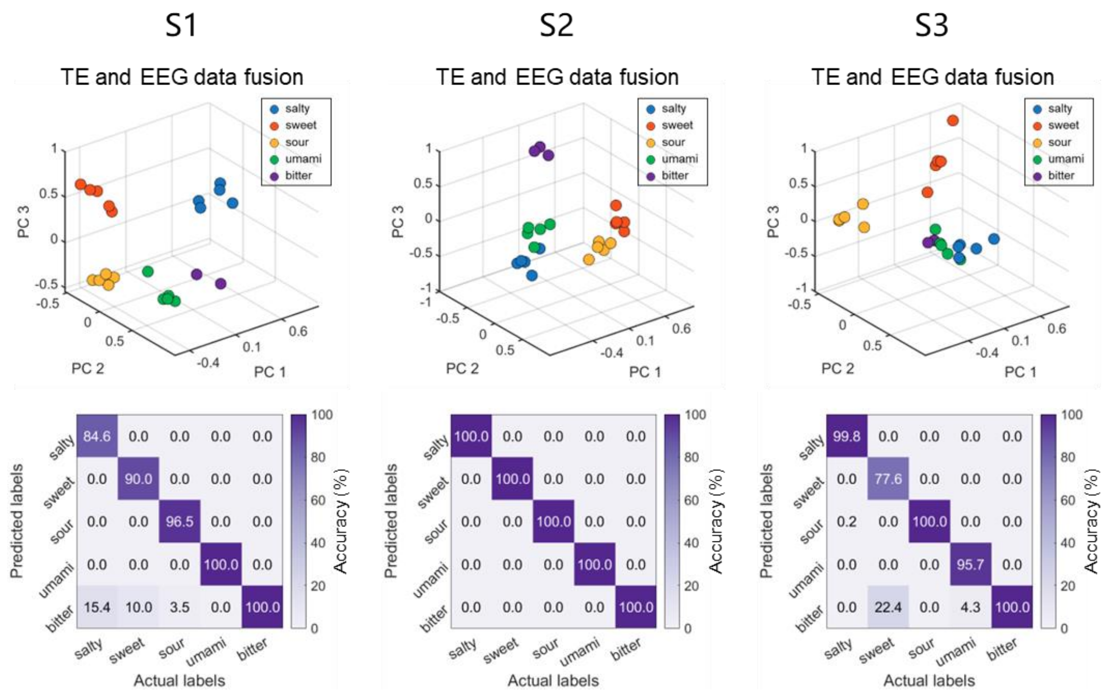

Supplementary Figure 29. Classification on individual subjects using fused data of TE and EEG signals. The top row shows the feature space of the modality of the fused TE and EEG data acquired from the three normal subjects, respectively. The classifier accuracy corresponding to each subject is shown in the confusion matrix of the bottom row, which suggests that dual-modal fusion can significantly enhance the accuracy of taste classification in each subject.

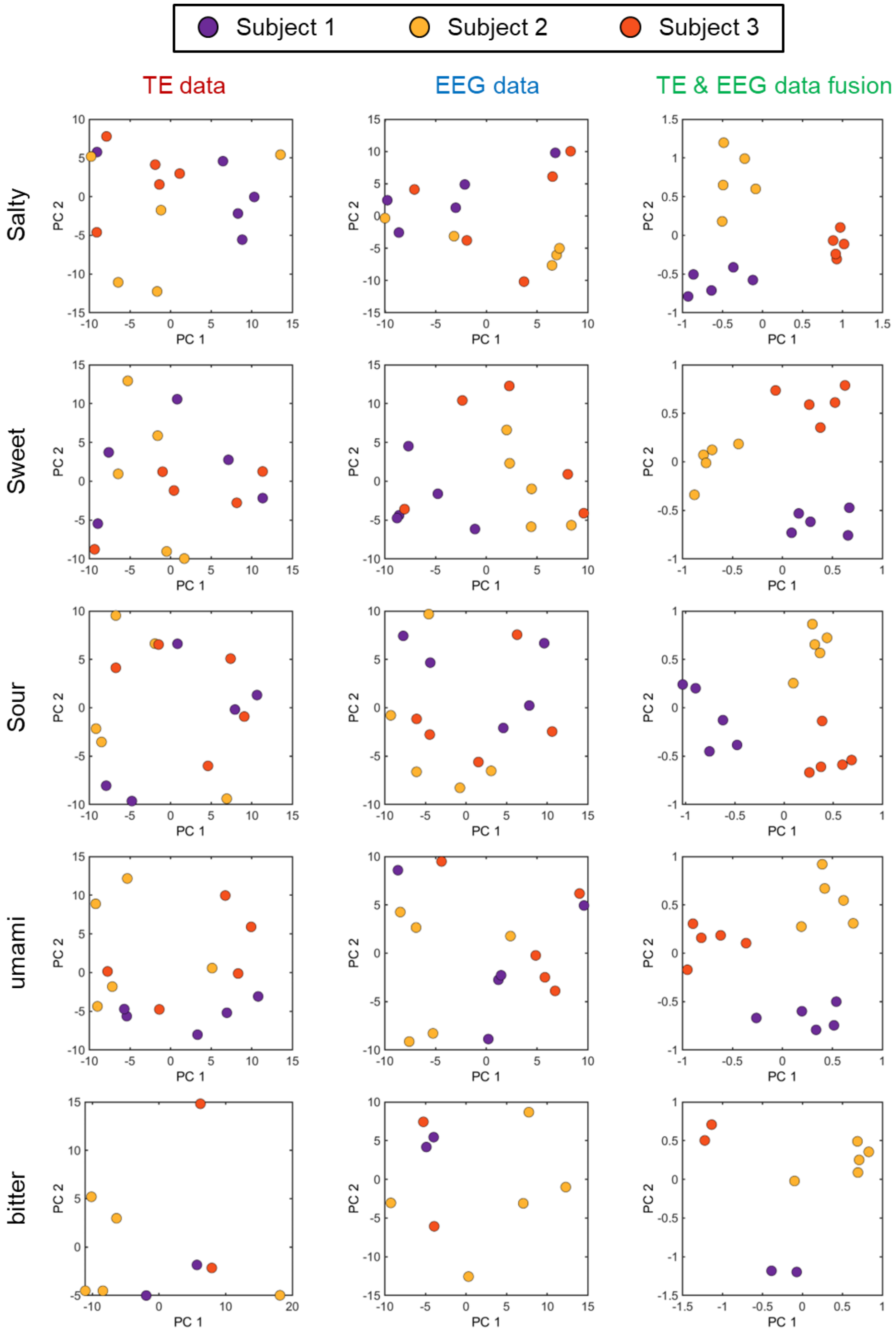

Supplementary Figure 30. Subject distinguishability under different taste stimulations using single and fused modalities. Each dot represents the corresponding information of the TE data, EEG data and dual-modal data in one trial.

By fusing the TE and EEG data, the points of the same subject are clustered together and different subjects can be differentiated.

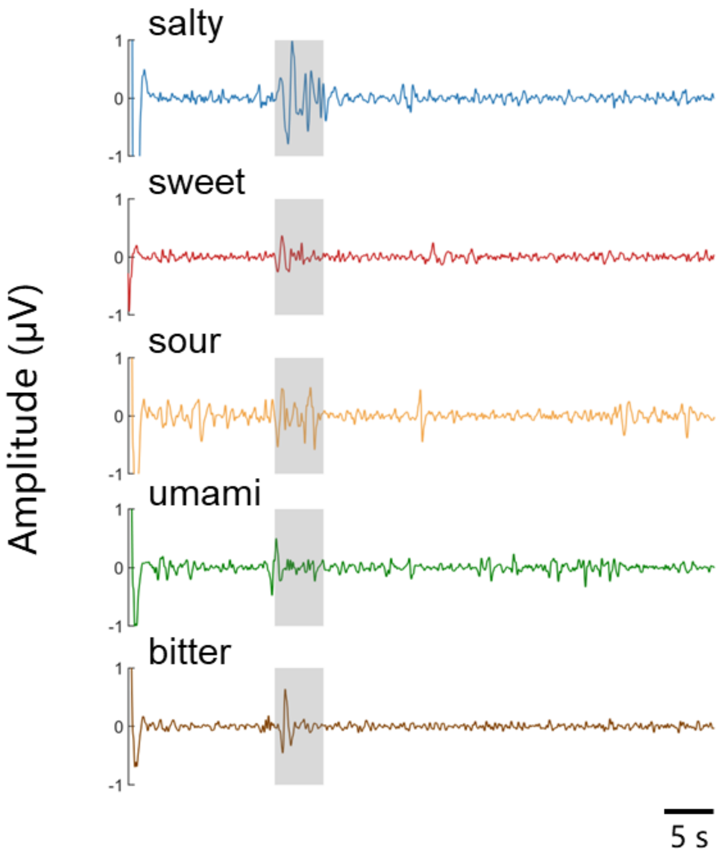

Supplementary Figure 31. EEG signals acquired from one patient with tongue cancer under taste stimulation. The filtered EEG signals for each taste stimulation are shown. The shaded grey areas indicate the events of taste stimulations.

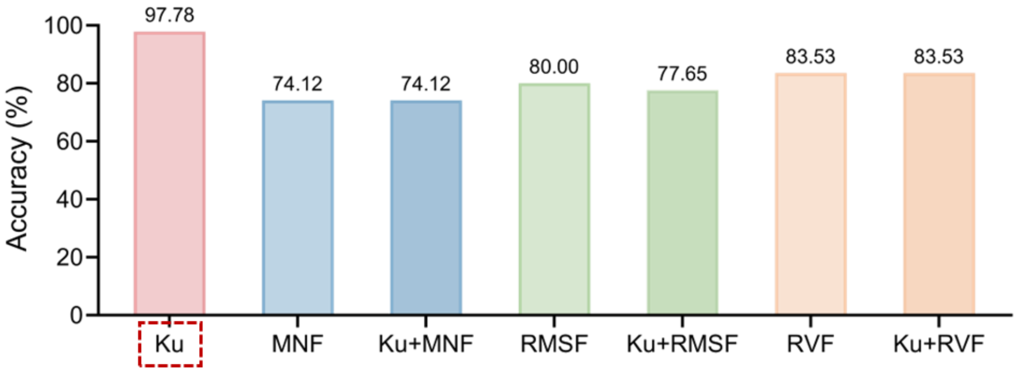

Supplementary Figure 32. Comparative analysis of cross-patient decoding accuracy on the gustatory information of the reconstructed tongue using time-domain, frequency-domain features and their combinations. The selected feature is outlined with dotted red lines.

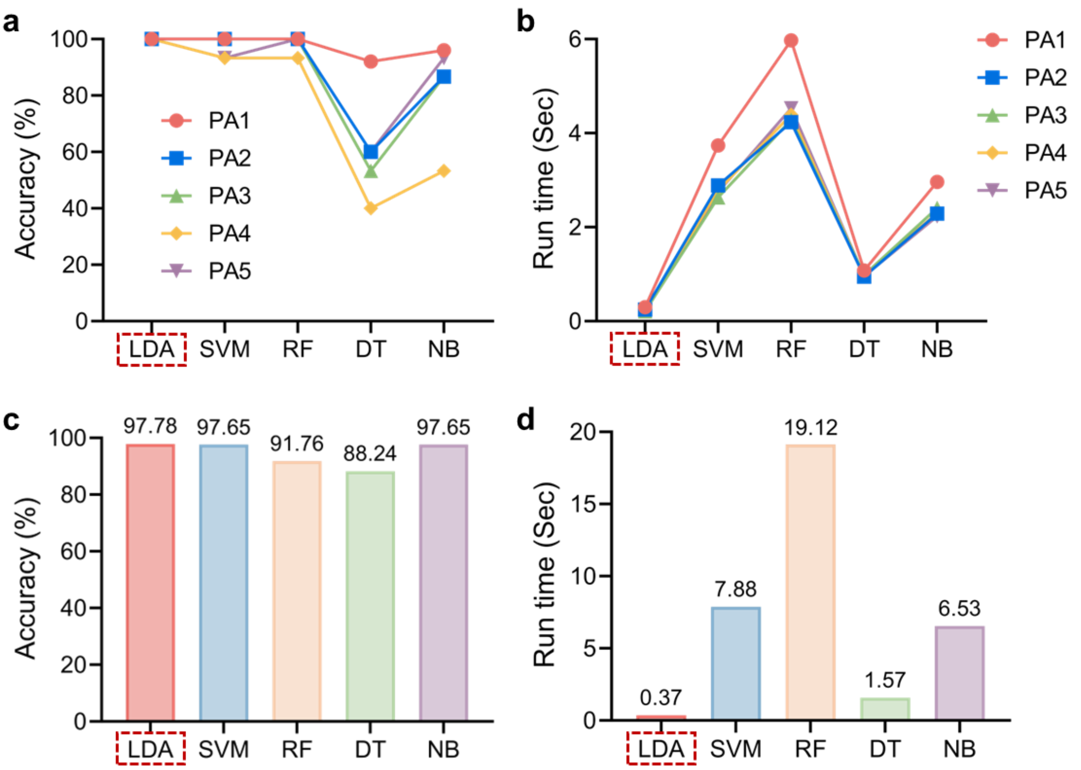

Supplementary Figure 33. Comparative analysis of performance metrics between different classifiers on individual patients and across patients. a, Decoding accuracy of the gustatory information from the reconstructed tongue utilizing different classifiers on individual patients (PA1, PA2, PA3, PA4, and PA5). **b,** Decoding time using different classifiers on individual patients. **c,** Cross-patient decoding accuracy of the gustatory information from the reconstructed tongue utilizing different classifiers. **d,** Decoding time using different classifiers on cross-patient datasets. The selected classifier is outlined with dotted red lines.

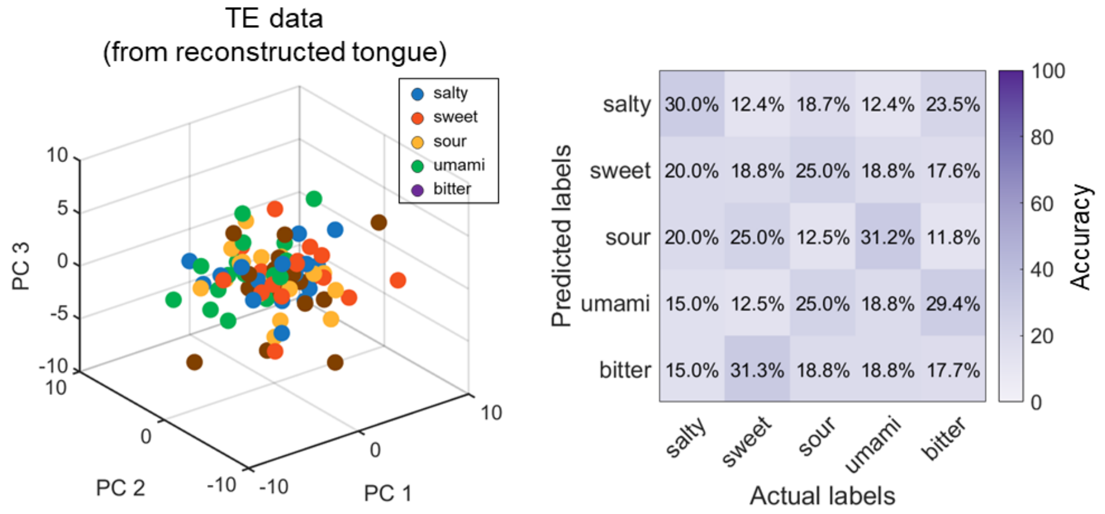

Supplementary Figure 34. Cross-patient decoding using TE data from the reconstructed tongue. The plot (left) shows the feature space of the TE data acquired from the reconstructed tongue of all five patients with tongue cancer. The final classifier accuracy using the single modality of the TE features is demonstrated in the confusion matrix (right).

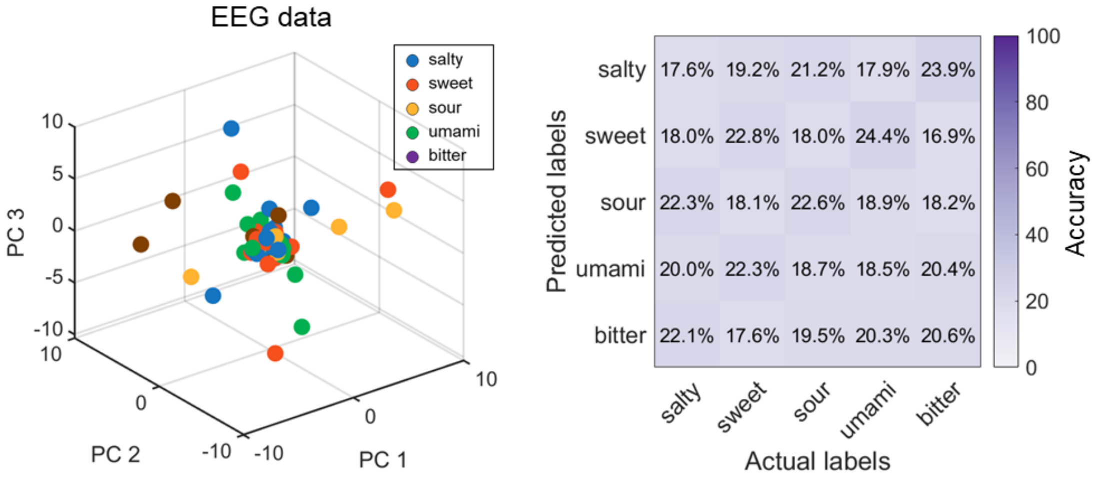

Supplementary Figure 35. Cross-patient decoding using EEG data. The plot (left) shows the feature space of the EEG data acquired from all five patients with tongue cancer. The final classifier accuracy using the single modality of the EEG features is demonstrated in the confusion matrix (right).

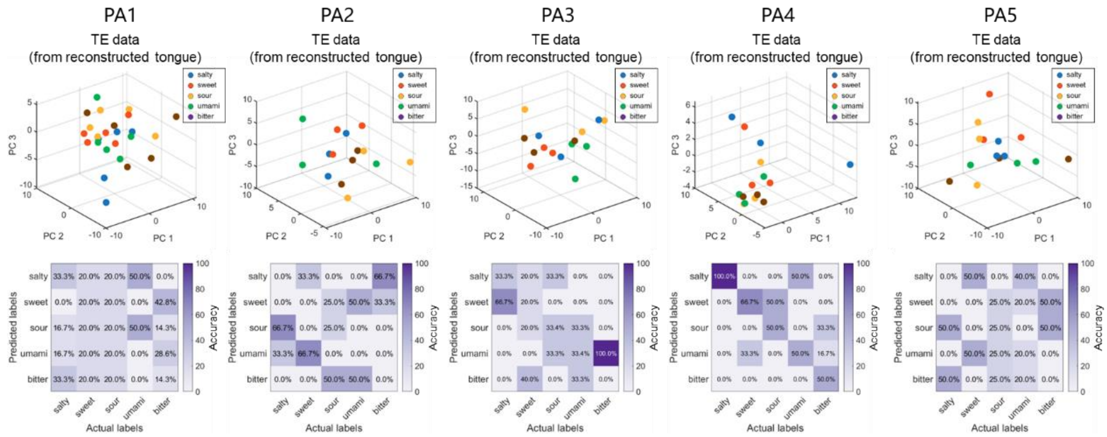

Supplementary Figure 36. Decoding on individual patients using TE data from the reconstructed tongue. The top row shows the feature space of the modality of TE data acquired from the reconstructed tongue in each of the five patients with tongue cancer. The classifier accuracy corresponding to each patient is shown in the confusion matrix of the bottom row.

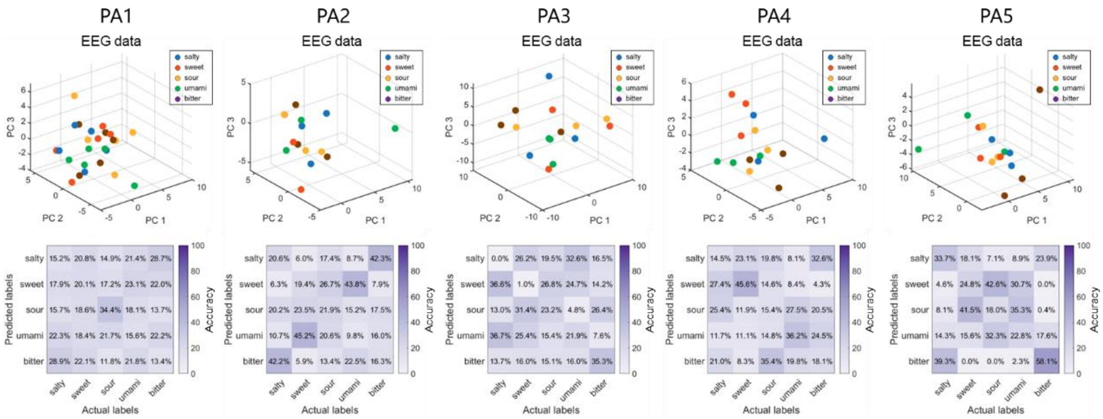

Supplementary Figure 37. Decoding on individual patients using EEG data. The top row shows the feature space of the modality of EEG data acquired from each of the five patients with tongue cancer. The classifier accuracy corresponding to each patient is shown in the confusion matrix of the bottom row.

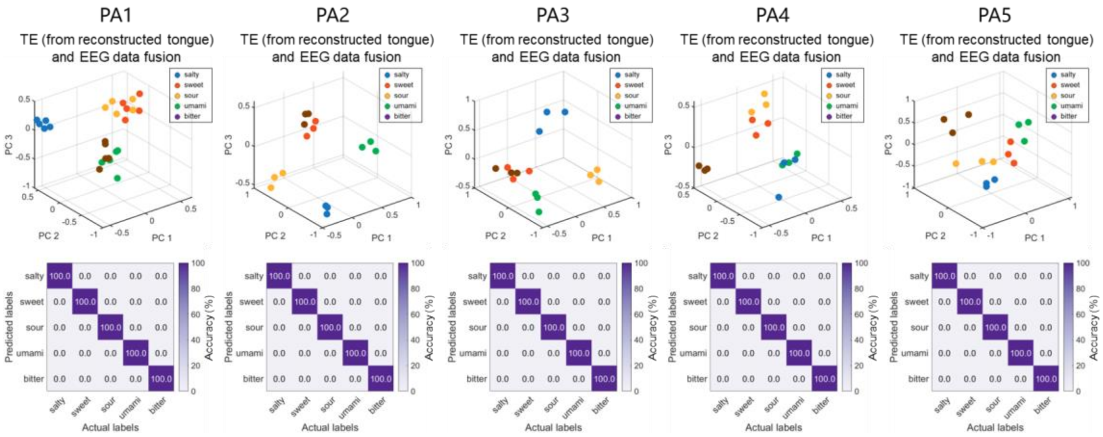

Supplementary Figure 38. Decoding on individual patients using fused data of the TE signals from the reconstructed tongue and EEG signals. The top row shows the feature space of the dual-modality after fusing TE data from the reconstructed tongue and EEG data in each of the five patients with tongue cancer. The classifier accuracy corresponding to each patient is shown in the confusion matrix of the bottom row.

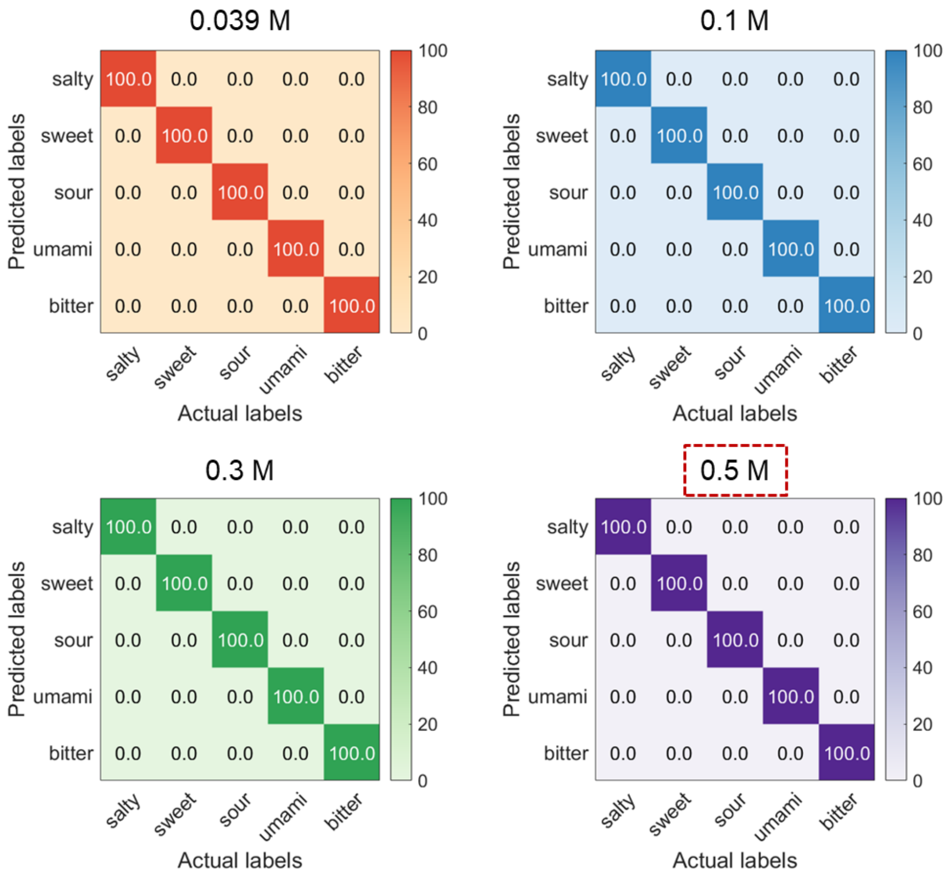

Supplementary Figure 39. Effects of taste stimuli solutions with different concentrations on decoding performance. Comparison of the decoding accuracy from four groups of taste stimuli solutions with different concentrations. Noted that the concentration of 0.039 M was adopted in the reference 34 and 35 for citric acid and 0.5 M was used in our manuscript.

Supplementary Table 1. Three investigated frequency-domain features.

No.	Frequency-domain Feature	Equation
1	Mean Frequency (MNF)	$\mathrm{MNF} = \frac{\sum_{j=1}^{M} f_j P_j}{\sum_{j=1}^{M} P_j}$
2	Root Mean Square Frequency (RMSF)	$\mathrm{RMSF} = \sqrt{\frac{\sum_{j=1}^{M} \left(f_j\right)^2 P_j}{\sum_{j=1}^{M} P_j}}$
3	Standard Variance Frequency (RVF)	$\mathrm{RVF} = \sqrt{\frac{\sum_{j=1}^{M} \left(f_j - f_c\right)^2 P_j}{\sum_{j=1}^{M} P_j}}$

Supplementary Table 2. Decoding accuracy using time-domain, frequency-domain features and their combinations on individual patients. The selected feature is outlined with red dotted lines.

	Ku	MNF	Ku+MNF	RMSF	Ku+RMSF	RVF	Ku+RVF
**PA1**	100	96.00	96.00	96.00	96.00	96.00	96.00
**PA2**	100	100	100	100	100	100	100
**PA3**	100	100	100	100	100	100	100
**PA4**	100	86.67	86.67	100	86.67	86.67	100
**PA5**	100	100	100	93.33	100	100	100

Note that Ku (Kurtosis) is the time-domain feature adopted in the original manuscript.
