# Supplementary material for: Gustatory interface for operative assessment and taste decoding in patients with tongue cancer
Source: Nat Commun. 2024 Oct 17;15:8967. doi: 10.1038/s41467-024-53379-y (PMC11487085; doi:10.1038/s41467-024-53379-y)
Supplement: Supplementary file 3 — Reporting summary [file 41467_2024_53379_MOESM3_ESM.pdf]

nature portfolio

Corresponding author(s): Zhitao Zhou

Last updated by author(s): Oct 6, 2024

Reporting Summary

Nature Portfolio wishes to improve the reproducibility of the work that we publish. This form provides structure for consistency and transparency in reporting. For further information on Nature Portfolio policies, see our Editorial Policies and the Editorial Policy Checklist.

Statistics

For all statistical analyses, confirm that the following items are present in the figure legend, table legend, main text, or Methods section.

n/a	Confirmed	
☐	☒	The exact sample size (n) for each experimental group/condition, given as a discrete number and unit of measurement
☐	☒	A statement on whether measurements were taken from distinct samples or whether the same sample was measured repeatedly
☐	☒	The statistical test(s) used AND whether they are one- or two-sided<br>*Only common tests should be described solely by name; describe more complex techniques in the Methods section.*
☐	☒	A description of all covariates tested
☐	☒	A description of any assumptions or corrections, such as tests of normality and adjustment for multiple comparisons
☐	☒	A full description of the statistical parameters including central tendency (e.g. means) or other basic estimates (e.g. regression coefficient) AND variation (e.g. standard deviation) or associated estimates of uncertainty (e.g. confidence intervals)
☐	☒	For null hypothesis testing, the test statistic (e.g. F, t, r) with confidence intervals, effect sizes, degrees of freedom and P value noted<br>*Give P values as exact values whenever suitable.*
☒	☐	For Bayesian analysis, information on the choice of priors and Markov chain Monte Carlo settings
☒	☐	For hierarchical and complex designs, identification of the appropriate level for tests and full reporting of outcomes
☒	☐	Estimates of effect sizes (e.g. Cohen's d, Pearson's r), indicating how they were calculated

Our web collection on statistics for biologists contains articles on many of the points above.

Software and code

Policy information about availability of computer code

Data collection	Tongue electrophysiological data was collected by a multichannel data acquisition system CereCube NSP8 (Neuroxess Co., Ltd., China). The EEG data were recorded using a gel-free electrode cap connected to the OpenBCI Cyton Biosensing Board (OpenBCI, NY, USA). CHI660E electrochemical workstation (CH Instruments Inc., China) for electrochemical data collection.
Data analysis	Matlab2021a for data analysis and plotting data. Origin 2020 and GraphPad Prism 10 for plotting data and data analysis. COMSOL Multiphysics 6.0 software for crosstalk simulation. The custom MATLAB scripts used for electrophysiology analysis are available from Zenodo (https://doi.org/10.5281/zenodo.13895193).

For manuscripts utilizing custom algorithms or software that are central to the research but not yet described in published literature, software must be made available to editors and reviewers. We strongly encourage code deposition in a community repository (e.g. GitHub). See the Nature Portfolio guidelines for submitting code & software for further information.

Data

Policy information about availability of data

All manuscripts must include a data availability statement. This statement should provide the following information, where applicable:

- Accession codes, unique identifiers, or web links for publicly available datasets
- A description of any restrictions on data availability
- For clinical datasets or third party data, please ensure that the statement adheres to our policy

All data supporting the findings of this study are available within the article and its supplementary files. Any additional requests for information can be directed to, and will be fulfilled by, the corresponding authors. Source data are provided with this paper.

Research involving human participants, their data, or biological material

Policy information about studies with human participants or human data. See also policy information about sex, gender (identity/presentation), and sexual orientation and race, ethnicity and racism.

Reporting on sex and gender	A total of seven subjects from Shanghai Ninth People's Hospital participated in this study (5 males and 2 females). Three normal and healthy subjects (2 males and 1 female) were also involved to validate the feasibility of the system.No sex- or gender-based analysis is used in this study.
Reporting on race, ethnicity, or other socially relevant groupings	In our study, we did not group participants according to race, ethnicity, or other socially relevant groupings.
Population characteristics	This research involved three patients diagnosed with tongue cancer who have not yet undergone surgery (age range: 45-60 years; 1 male, 2 females), along with five patients who had undergone surgery of tongue resection and transplantation (age range: 45-70 years; 5 males). One of the patients met the predetermined inclusion criteria both before and after the surgical intervention, who was diagnosed with primary tongue cancer and underwent subsequent tongue flap resection and reconstruction surgery. Three postoperative patients underwent two follow-up examinations to monitor the recovery using tongue electrical signals.
Recruitment	Clinical participants were enrolled from the out-patients clinic of oral and maxillofacial surgery who must fit specific clinical characteristics. Specifically, they have been diagnosed with primary tongue cancer or have undergone resection and reconstructive surgery, while excluding interfering factors such as inflammation and medication. The participants were informed that their decision to participate in the research study was strictly voluntary, and would not impact their clinical treatment. Therefore, we do not expect any noteworthy self-selection bias in this study, as participants who volunteer to participate will not differ from non-volunteers in any relevant clinical characteristics.
Ethics oversight	The study received ethical approval from the ShaInstitutional Review Board of Shanghai Jiao Tong University School of Medicine (Approval No.:SH9H-2023-T174-2) and was conducted according to the guidelines of all relevant ethical regulations.

Note that full information on the approval of the study protocol must also be provided in the manuscript.

Field-specific reporting

Please select the one below that is the best fit for your research. If you are not sure, read the appropriate sections before making your selection.

☒ Life sciences ☐ Behavioural & social sciences ☐ Ecological, evolutionary & environmental sciences

For a reference copy of the document with all sections, see nature.com/documents/nr-reporting-summary-flat.pdf

Life sciences study design

All studies must disclose on these points even when the disclosure is negative.

Sample size	Seven subjects from the Shanghai Ninth People's Hospital including 5 males and 2 females and three normal and healthy subjects including 2 males and 1 female were recruited for this study. The amount of data collected with the participant needs to meet the requirement to reasonably estimate the measurements of interest and perform statistical comparisons and also depends on the amount of time each participant was willing to volunteer for the study. We consider our sample size is sufficient to investigate the effects we observe.
Data exclusions	No data were excluded from analysis.
Replication	We conducted experiments across seven participants, with each subject performing 3-5 repetitions under each taste stimulation. Across patients, the high decoding performance was consistent between patients that demonstrates reproducibility of results.
Randomization	According to the diagnosis and treatment stage of the participants and the purpose of analysis, participants were allocated into preoperative or postoperative groups while the taste stimulation experiments were conducted in a random fashion. The five taste stimuli were provided in a random order.

Blinding	The investigators were not blinded to allocation during experiment and outcome assessment as the analysis of electrophysiological signals caused by tongue cancer lesions or reconstructive surgery cannot be done without knowledge of the basic condition of the participants. We ensure that the data used to train models remained separate from the test data. For each participant, the data was split based on the collection site into training and testing datasets.

Reporting for specific materials, systems and methods

We require information from authors about some types of materials, experimental systems and methods used in many studies. Here, indicate whether each material, system or method listed is relevant to your study. If you are not sure if a list item applies to your research, read the appropriate section before selecting a response.

Materials & experimental systems

n/a	Involved in the study
☒	☐ Antibodies
☒	☐ Eukaryotic cell lines
☒	☐ Palaeontology and archaeology
☒	☐ Animals and other organisms
☒	☐ Clinical data
☒	☐ Dual use research of concern
☒	☐ Plants

Methods

n/a	Involved in the study
☒	☐ ChIP-seq
☒	☐ Flow cytometry
☒	☐ MRI-based neuroimaging

Plants

Seed stocks	This is not relevant to our study.
Novel plant genotypes	This is not relevant to our study.
Authentication	This is not relevant to our study.
